# Nutlet micromorphology of Japanese *Salvia* (Salviinae, Lamiaceae) and its systematic significance

**DOI:** 10.3897/phytokeys.279.199134

**Published:** 2026-09-11

**Authors:** Amjad Khan, Atsuko Takano, Chung-Kun Lee, Hiroaki Ishida

**Affiliations:** 1 Graduate School of Human Science and Environment, University of Hyogo,1-1-2 Shinzaikehonmachi, Himeji, 670-0092, Japan Graduate School of Human Science and Environment, University of Hyogo Himeji Japan; 2 Institute of Natural & Environmental Sciences, University of Hyogo, 6 chome, Yayoigaoka, Sanda, 669-1546, Japan Institute of Natural & Environmental Sciences, University of Hyogo Sanda Japan; 3 Museum of Nature and Human Activities, Hyogo, 6 chome Yayoigaoka, Sanda, 669-1546, Japan Museum of Nature and Human Activities Sanda Japan

**Keywords:** Japan, nutlet morphology, *
Salvia
*, SEM, taxonomy

## Abstract

*Salvia* is a taxonomically challenging genus in the Japanese archipelago, comprising 12 distinct species, eight of which are endemic. In this study, the nutlet micromorphology of 19 taxa (species and varieties) assigned to the sections *Glutinaria*, *Heterosphace*, *Notiosphace*, and *Sobiso* was investigated using light and scanning electron microscopy. This study aimed to evaluate the systematic significance of nutlet morphology in Japanese *Salvia*. Nutlet size ranged from 0.64 × 0.45 mm in *S. plebeia* (sect. *Notiosphace*) to 3.27 × 2.27 mm in *S. koyamae* (sect. *Glutinaria*). The L/W ratio varied from 1.06 in S. glabrescens var. repens (sect. *Glutinaria*) to 2.54 in S. pygmaea var. pygmaea (sect. *Sobiso*). Nutlet shapes were elliptic, narrow-elliptic, and ellipsoidal in sect. *Sobiso*; ovoid to obovoid and spherical in sect. *Glutinaria*; and ovoid in both sect. *Heterosphace* and sect. *Notiosphace*. Abscission scar morphology varied among the sections, being squarish, triangular, irregular, or orbicular in sect. *Sobiso*; irregular to orbicular in sect. *Glutinaria*; orbicular in sect. *Heterosphace*; and triangular in sect. *Notiosphace*. Four major types of surface sculpturing were recognized: colliculate with irregular surface cells in sect. *Glutinaria*; reticulate with pentagonal to hexagonal cells in sect. *Sobiso*; tuberculate with large rounded knobs in sect. *Heterosphace*; and verrucate with small wart-like projections in sect. *Notiosphace*. Principal component analysis (PCA) based on taxon means demonstrated that nutlet size-related characters contributed most strongly to the observed variation among the studied taxa. Overall, the diversity of nutlet morphology highlights the diagnostic value of these characters for taxonomic identification at both the species and sectional levels within Japanese *Salvia*.

## Introduction

*Salvia* L. is a species-rich and easily recognizable genus within the subtribe Salviinae (tribe Mentheae, subfamily Nepetoideae, family Lamiaceae), comprising approximately 1,088 species (Kriebel et al. 2019; Drew 2020; POWO 2025). The genus has an almost cosmopolitan distribution (Hu et al. 2018). *Salvia* has undergone significant species radiations in three global biodiversity hotspots: the Americas (Central to South America, with 510 species, and North America, with 94 species), followed by Asia (270 species in Western Asia and 97 species in East Asia), and Europe, hosting 117 species (Wei et al. 2015; Rose et al. 2021). At the country level, Mexico harbors the highest number of species (322), followed by Russia (109), Turkey (88), the United States (85), and China (82) (Wei et al. 2015; POWO 2025).

This study focuses on the genus *Salvia* in Japan, which comprises 12 species (Murata and Yamazaki 1993; Inoue 1997; Inoue and Ozawa 1998; Hihara et al. 2001; Takano and Okada 2011; Takano et al. 2014; Hu et al. 2018; Hayakawa et al. 2024). Among the 12 *Salvia* species, eight are endemic, while the remaining four have broader distributions (Takano et al. 2014; Hu et al. 2018; Hayakawa et al. 2024). These include *S. japonica* Thunb., also found in Korea, Taiwan, and China; *S. nipponica* Miq., present in both China and Japan; and *S. plebeia* R. Br., which is widely distributed across Asia and Australia (Murata and Yamazaki 1993; Hu et al. 2018). Most recently, Hayakawa et al. (2024) reported the first naturalization of *Salvia lyrata* L. from a roadside on Niijima Island, Izu Islands, Japan. This species, native to the United States (Marín et al. 1996; POWO 2025), is cultivated as a garden herb in Japan and has shown the potential to escape from cultivation and become naturalized in the wild (Hayakawa et al. 2024).

Japanese *Salvia* has traditionally been classified into three subgenera: subg. *Salvia*, subg. *Sclarea* Mill., and subg. *Allagospadonopsis* Briq. (Wu 1977; Murata and Yamazaki 1993). Most recently, based on molecular and morphological evidence, Hu et al. (2018) reclassified all East Asian *Salvia* into a newly recognized subgenus, *Glutinaria* (Raf.) G.X. Hu, C.L. Xiang & B.T. Drew, and further recognized eight sections within this subgenus. All native Japanese *Salvia* species were placed in subg. *Glutinaria* and assigned to three sections. Sect. *Sobiso* (Raf.) G.X. Hu, A. Takano & B.T. Drew comprises *Salvia akiensis* A. Takano, Sera & N. Kurosaki, *S. isensis* Nakai ex H. Hara, *S. japonica* Thunb., *S. lutescens* (Koidz.) Koidz., *S. omerocalyx* Hayata, *S. pygmaea* Matsum., and *S. ranzaniana* Makino. Sect. *Glutinaria* (Raf.) G.X. Hu, C.L. Xiang & B.T. Drew includes *S. koyamae* Makino, *S. glabrescens* Makino, *S. nipponica* Miq., and *Salvia × sakuensis* Naruh. & Hihara, whereas sect. *Notiosphace* Benth. includes *S. plebeia* R. Br. Furthermore, the recently introduced *S. lyrata* was originally placed in sect. *Heterosphace* Benth. (Benth. & Hook.f., Gen. Pl. 2: 1196, 1876), within subgenus *Leonia* Benth., a subgeneric name later noted as invalidly published (Marín et al. 1996; Govaerts 2003; Kalnyuk et al. 2025). However, recent molecular phylogenetic studies instead place *S. lyrata* within an informally named “*Heterosphace*” clade (Kriebel et al. 2019; Rose et al. 2021), treated by some authors as an unranked subgenus pending formal validation.

Previous studies have demonstrated the taxonomic significance of nutlet morphology in several *Salvia* taxa (Ryding 2001; Özkan et al. 2009; Ifrim 2012). In addition to its importance for species identification, nutlet morphology has increasingly been recognized as an important source of taxonomic information supporting existing sectional circumscriptions within *Salvia* (Oran 1997; Kahraman et al. 2009; Kahraman 2011; Polat et al. 2015; El-Rabiai and Elfaidy 2021). Nutlet characters, including size, shape, surface ornamentation, and abscission scar morphology, provide relatively conservative and independent morphological evidence that complements the floral and staminal characters traditionally used for taxonomic circumscription of sections and subgenera of *Salvia* (Özkan et al. 2009; Kahraman et al. 2010a, 2010b; Büyükkartal et al. 2011; Ifrim 2012; Ananthalakshmi 2020). Although Bentham’s (1876) traditional classification of Old World *Salvia* was primarily based on staminal and corolla morphology, subsequent studies have demonstrated that nutlet morphological characters, particularly surface ornamentation, correspond closely with sectional boundaries and therefore have considerable systematic importance (Oran 1996; Kahraman 2011; Mousavi et al. 2013; Celep et al. 2014; Alimpić et al. 2021). Among these characters, nutlet surface ornamentation has consistently been identified as one of the most informative diagnostic features for sectional delimitation in *Salvia* (Oran 1996; Kahraman 2011; Salimpour et al. 2014; Kılıç and Kılıç 2021). The nutlet morphology of Japanese *Salvia* remains poorly documented despite its systematic importance (Li et al. 2016). To date, only two species, *S. lyrata* and *S. plebeia*, have been investigated (Marín et al. 1996; Li et al. 2016), both of which are also examined in the present study. A detailed investigation of nutlet morphology across the Japanese taxa is therefore necessary to assess the systematic significance of these characters.

The present study describes the nutlet morphology of 19 taxa (species and varieties) of Japanese *Salvia*, representing four sections: *Glutinaria*, *Heterosphace*, *Notiosphace*, and *Sobiso*. Nutlet micromorphological characters were investigated to evaluate their taxonomic utility and systematic significance for species identification at the sectional level. Specifically, the present study aims to (a) document the variation in nutlet morphology among the examined taxa; (b) assess whether nutlet micromorphological characters provide reliable information for the current sectional circumscription of Japanese *Salvia*; and (c) classify nutlet surface ornamentation into distinct descriptive categories and evaluate their taxonomic significance for existing sectional classifications.

## Materials and methods

### Study species and resources

**Figure 1.**
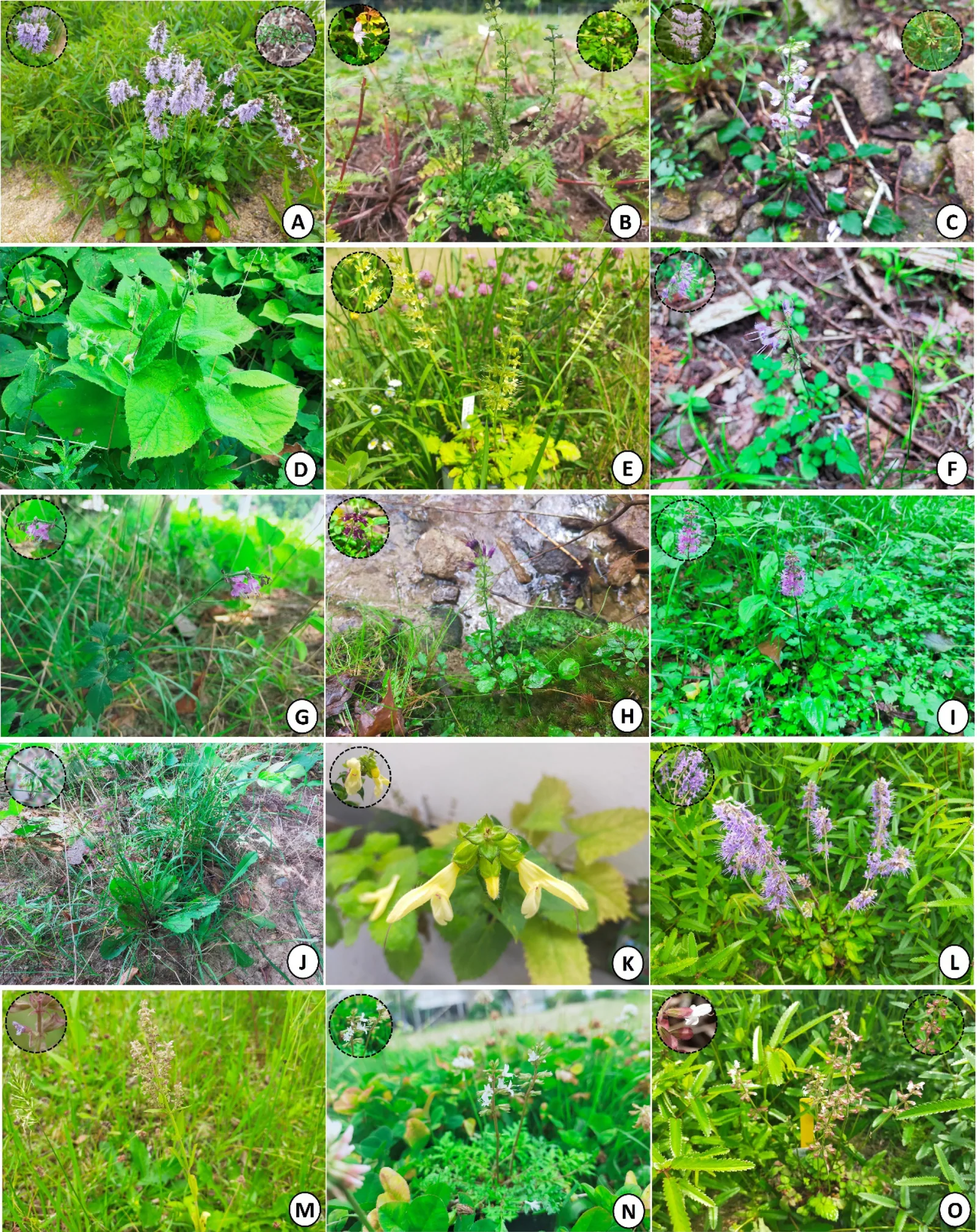
Field photographs of some Japanese *Salvia*: **A**. *S. akiensis*; **B**. *S. isensis*; **C**. *S. japonica*; **D**. *S. koyamae*; **E**. S. lutescens var. lutescens; **F**. S. lutescens var. crenata; **G**. S. lutescens var. intermedia; **H**. S. lutescens var. occidentalis; **I**. S. lutescens var. stolonifera; **J**. *S. lyrata*; **K**. *S. nipponica*; **L**. S. omerocalyx var. omerocalyx; **M**. *S. plebeia*; **N**. S. pygmaea var. pygmaea; **O**. *S. ranzaniana*.

**Figure 2.**
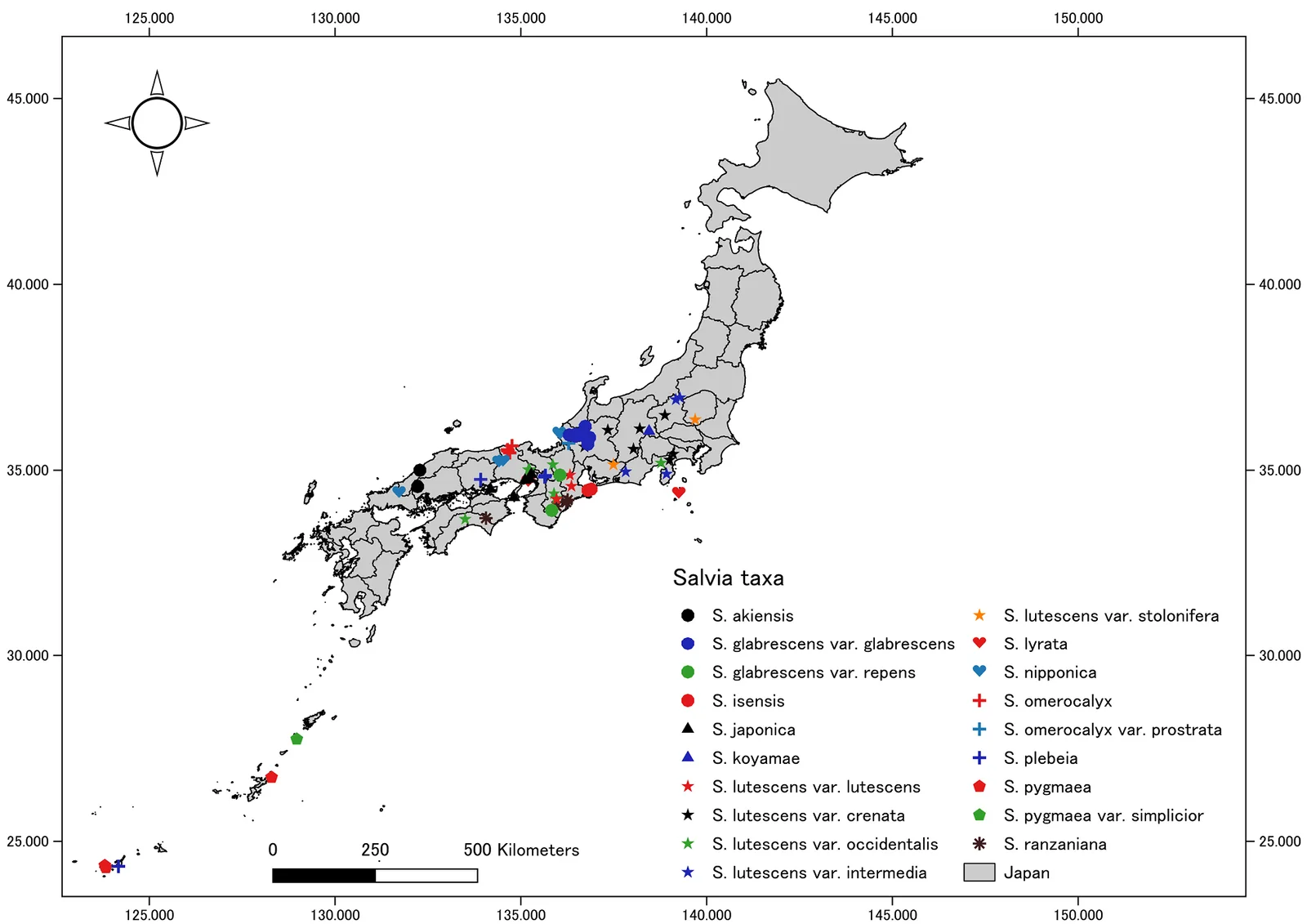
Map showing the distribution of *Salvia* taxa used in this study.

Mature nutlets were detached from specimens housed at the HYO and KYO herbaria, following acronyms established by Thiers (2025). A total of 84 specimens, representing 19 *Salvia* taxa (species and varieties), were examined. Field photographs were provided for some taxa (Fig. 1). Voucher information, including localities, collector details, and other field data, is provided in Appendix 1. Taxon names were cross-checked using the International Plant Names Index (IPNI 2025), and data on distribution ranges were obtained from Plants of the World Online (POWO 2025). A distribution map of Japan was generated based on the herbarium specimen records (Fig. 2).

### Microscopy (LM and SEM) and terminology

Nutlet gross morphology, including shape, color, surface ornamentation, and abscission scar characteristics, was examined and photographed using a digital camera (Olympus, BJ5C 42363) connected to a binocular stereomicroscope (Nikon, C-PSN 1014452). Additionally, numerical traits were recorded from 5–40 nutlet samples per specimen for each taxon, with measurements taken using a microscope (WRAYMER HM20L1712001). Size measurements, including nutlet length, nutlet width, abscission scar length, abscission scar width, and L/W ratio, were assessed following the terminology of Mousavi et al. (2013), Salimpour et al. (2014), and Kılıç and Kılıç (2021).

For scanning electron microscope (SEM) analysis, mature nutlets were selected, and surface debris was removed using alcohol (70% ethanol). The air-dried nutlets were mounted on aluminum stubs using double-sided adhesive tape and coated with gold using a JEOL JEC-550. Subsequently, the surface sculpturing of the nutlets was examined and photographed using a KEYENCE scanning electron microscope (Model VE-9800).

Nutlet shapes were assessed from both dorsal and ventral perspectives. The dorsal surface was used to characterize surface sculpturing patterns, while the ventral surface was examined to determine the position of the abscission scar. Terminology for describing nutlet morphological features follows Ryding (1995) and Marín et al. (1996). Nutlet shapes were identified based on overall planar or two-dimensional form, as standardized by Stearn (2004) and Simpson (2006).

### Data analyses

Numerical data and descriptive statistics (arithmetic average, maximum and minimum values [max and min], and standard deviation [±SD]) were calculated using SPSS (version 16.0) and are summarized in Table 1. Furthermore, principal component analysis (PCA) was performed to assess morphological variation and to examine relationships among the studied *Salvia* taxa based on the mean values of nutlet quantitative characters, following Van Emden (2019). The analysis was conducted using R (version 4.0.4), following R Core Team (2021), with all variables standardized prior to analysis (scale = TRUE) to account for differences in measurement units and scales. The dataset comprised six quantitative nutlet characters recorded for each taxon, and *Salvia* taxa were treated as grouping factors. The first two principal components (PC1 and PC2) were used to visualize the ordination of taxa in a two-dimensional scatter plot. The percentage of variance explained by each principal component was calculated to evaluate the relative significance of each axis.

**Table 1.** Nutlet micromorphological (quantitative) characteristics of Japanese *Salvia*.

**Taxon name**	**Nutlet length**	**Nutlet width**	**L/W ratio**	**Abscission scar length**	**Abscission scar width**	**L/W ratio**
	**max−min; M ± SD (mm)**	**max–min; M ± SD (mm)**		**max–min; M ± SD (mm)**	**max−min; M ± SD (mm)**	
***S. akiensis*** A. Takano, Sera & N. Kurosaki	1.50−1.10 = 1.30 ± 0.14	1.00−0.50 = 0.75 ± 0.18	1.73	0.03−0.02 = 0.025 ± 0.00	0.03−0.02 = 0.021 ± 0.00	1.19
***S. isensis*** Nakai ex H. Hara	1.60−1.10 = 1.30 ± 0.16	0.70−0.40 = 0.53 ± 0.12	2.45	0.03−0.02 = 0.025 ± 0.00	0.03−0.01 = 0.023 ± 0.00	1.08
***S. japonica*** Thunb.	2.00−1.00 = 1.26 ± 0.31	1.00−0.50 = 0.75 ± 0.13	1.68	0.30−0.02 = 0.056 ± 0.07	0.30−0.01 = 0.039 ± 0.06	1.43
***S. lutescens*** (Koidz.) Koidz. **var. lutescens**	2.00−1.20 = 1.74 ± 0.20	1.00−0.50 = 0.75 ± 0.11	2.32	0.04−0.02 = 0.027 ± 0.00	0.05−0.01 = 0.026 ± 0.00	1.03
**S. lutescens var. occidentalis** A. Takano	2.10−1.40 = 1.68 ± 0.22	1.20−0.60 = 0.90 ± 0.15	1.86	0.04−0.02 = 0.029 ± 0.00	0.04−0.01 = 0.026 ± 0.00	1.11
**S. lutescens var. crenata** (Makino) Murata	2.00−1.20 = 1.79 ± 0.20	1.00−0.50 = 0.86 ± 0.12	2.08	0.04−0.02 = 0.033 ± 0.00	0.04−0.02 = 0.032 ± 0.00	1.03
**S. lutescens var. stolonifera** G. Nakai	1.90−1.00 = 1.69 ± 0.24	1.00−0.60 = 0.80 ± 0.13	2.11	0.04−0.02 = 0.029 ± 0.00	0.04−0.02 = 0.029 ± 0.00	1.00
**S. lutescens var. intermedia** (Makino) Murata	2.00−1.20 = 1.66 ± 0.23	0.90−0.70 = 0.83 ± 0.07	2.00	0.04−0.03 = 0.034 ± 0.00	0.04−0.02 = 0.031 ± 0.00	1.09
***S. omerocalyx*** Hayata **var. omerocalyx**	2.00−1.40 = 1.68 ± 0.19	1.10−0.80 = 0.96 ± 0.09	1.75	0.07−0.04 = 0.053 ± 0.00	0.07−0.04 = 0.054 ± 0.00	1.01
**S. omerocalyx var. prostrata** Satake	2.20−1.80 = 2.00 ± 0.11	1.30−1.20 = 1.26 ± 0.03	1.58	0.08−0.07 = 0.073 ± 0.03	0.07−0.06 = 0.066 ± 0.03	1.10
***S. pygmaea*** Matsum. **var. pygmaea**	1.60−1.00 = 1.35 ± 0.20	0.70−0.40 = 0.54 ± 0.08	2.54	0.03−0.01 = 0.021 ± 0.00	0.03−0.01 = 0.022 ± 0.00	0.95
**S. pygmaea var. simplicior** Hatus. ex T. Yamaz.	1.60−1.50 = 1.56 ± 0.05	0.70−0.60 = 0.63 ± 0.05	2.47	0.03−0.02 = 0.026 ± 0.00	0.03−0.02 = 0.023 ± 0.00	1.13
***S. ranzaniana*** Makino	2.50−1.40 = 2.10 ± 0.41	1.80−0.70 = 1.22 ± 0.36	1.72	0.06−0.03 = 0.048 ± 0.00	0.06−0.03 = 0.047 ± 0.00	1.02
***S. lyrata*** L.	2.10−1.80 = 1.91 ± 0.09	2.10−1.50 = 1.78 ± 0.18	1.07	0.04−0.02 = 0.031 ± 0.00	0.04−0.02 = 0.032 ± 0.00	0.96
***S. koyamae*** Makino	3.50−3.10 = 3.27 ± 0.17	2.60−2.00 = 2.27 ± 0.27	1.44	0.09−0.07 = 0.080 ± 0.00	0.09−0.07 = 0.080 ± 0.00	1.00
***S. nipponica*** Miq.	2.60−2.00 = 2.32 ± 0.20	2.10−1.60 = 1.92 ± 0.15	1.20	0.08−0.05 = 0.064 ± 0.00	0.07−0.05 = 0.062 ± 0.00	1.03
***S. glabrescens*** Makino **var. glabrescens**	3.50−1.80 = 2.33 ± 0.40	3.20−1.70 = 2.12 ± 0.38	1.09	0.08−0.04 = 0.060 ± 0.01	0.07−0.03 = 0.050 ± 0.00	1.20
**S. glabrescens var. repens** (Koidz.) S. Kurosaki	2.30−1.00 = 1.95 ± 0.27	2.10−1.50 = 1.83 ± 0.17	1.06	0.05−0.03 = 0.042 ± 0.00	0.05−0.03 = 0.030 ± 0.00	1.40
***S. plebeia*** R. Br.	0.80−0.50 = 0.64 ± 0.10	0.60−0.30 = 0.45 ± 0.07	1.42	0.01−0.01 = 0.010 ± 0.00	0.01−0.01 = 0.010 ± 0.00	1.00

## Results

**Figure 3.**
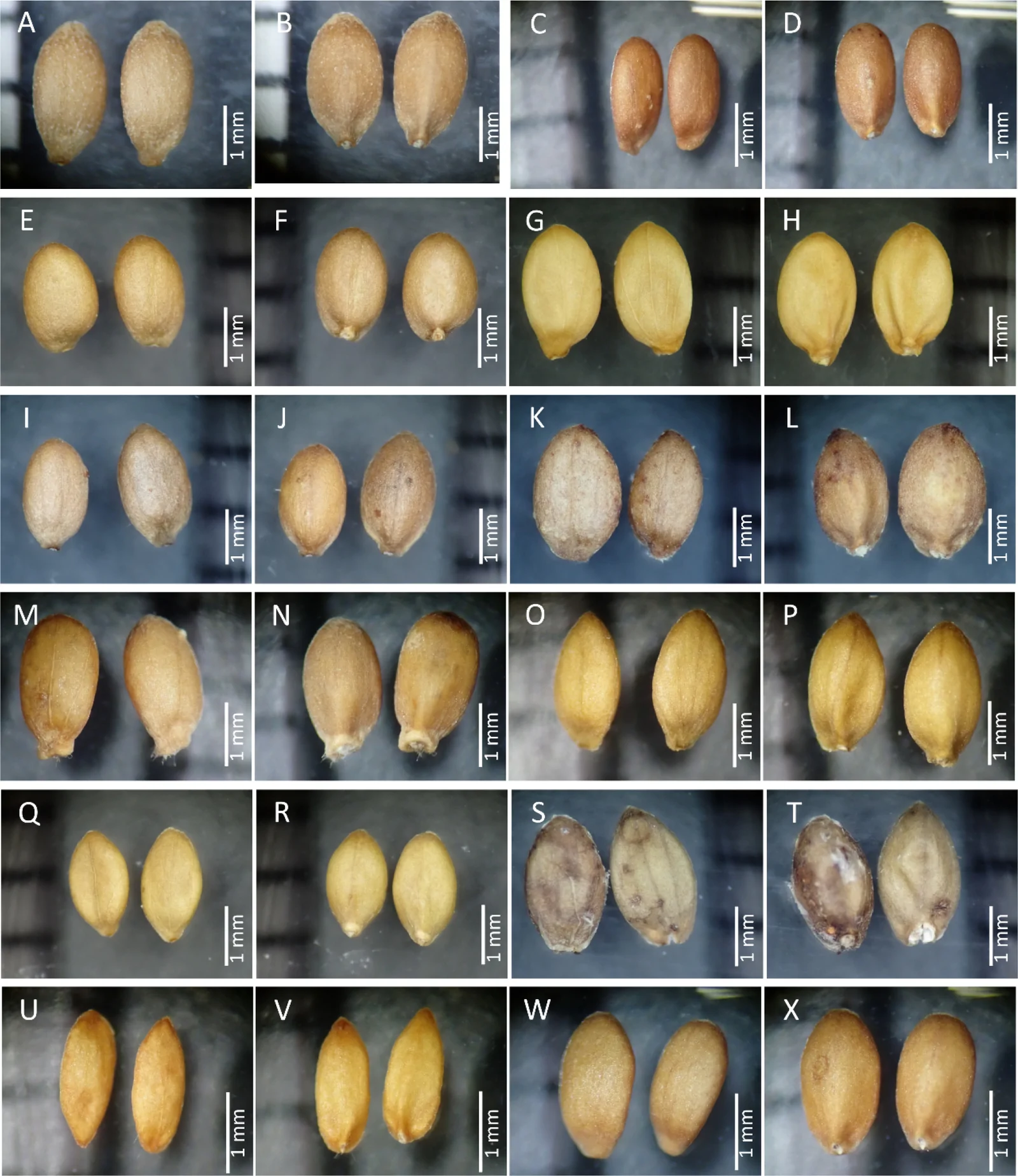
LM micrographs showing nutlets of *Salvia* sect. *Sobiso*: **A, C, E, G, I, K, M, O, Q, S, U, W**. Dorsal views of nutlets; **B, D, F, H, J, L, N, P, R, T, V, X**. Ventral views of nutlets. Species: **A, B**. *S. akiensis*; **C, D**. *S. isensis*; **E, F**. *S. japonica*; **G, H**. S. lutescens var. lutescens; **I, J**. S. lutescens var. crenata; **K, L**. S. lutescens var. intermedia; **M, N**. S. lutescens var. occidentalis; **O, P**. S. lutescens var. stolonifera; **Q, R**. S. omerocalyx var. omerocalyx; **S, T**. S. omerocalyx var. prostrata; **U, V**. S. pygmaea var. pygmaea; **W, X**. S. pygmaea var. simplicior.

**Figure 4.**
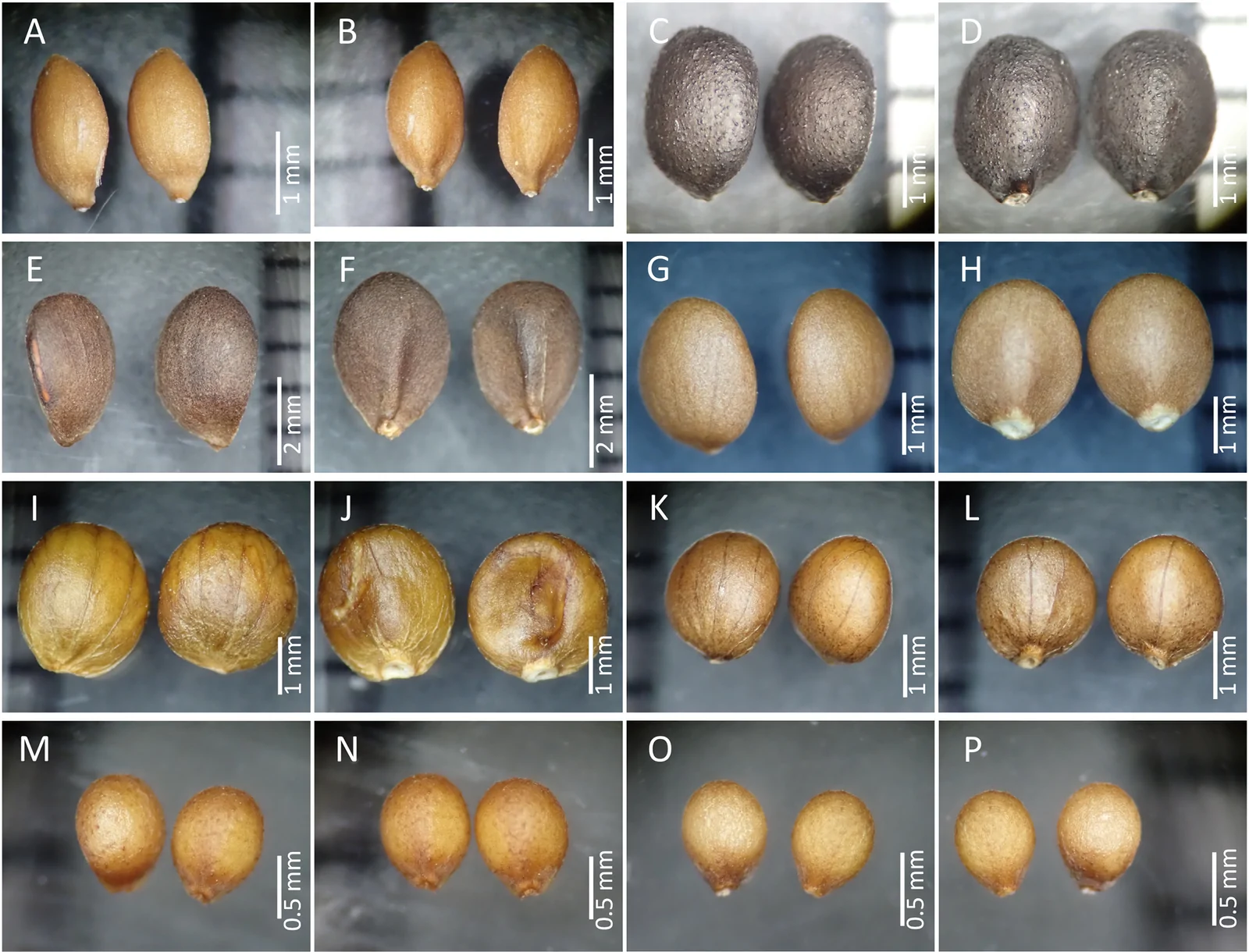
LM micrographs showing nutlet shape in *Salvia* species: **A, C, E, G, I, K, M, O**. Dorsal views of nutlets; **B, D, F, H, J, L, N, P**. Ventral views of nutlets. Sections: **A, B**. Sect. *Sobiso*; **C, D**. Sect. *Heterosphace*; **E–L**. Sect. *Glutinaria*; **M–P**. Sect. *Notiosphace*. Species: **A, B**. *S. ranzaniana*; **C, D**. *S. lyrata*; **E, F**. *S. koyamae*; **G, H**. *S. nipponica*; **I, J**. S. glabrescens var. glabrescens; **K, L**. S. glabrescens var. repens; **M–P**. *S. plebeia*.

**Figure 5.**
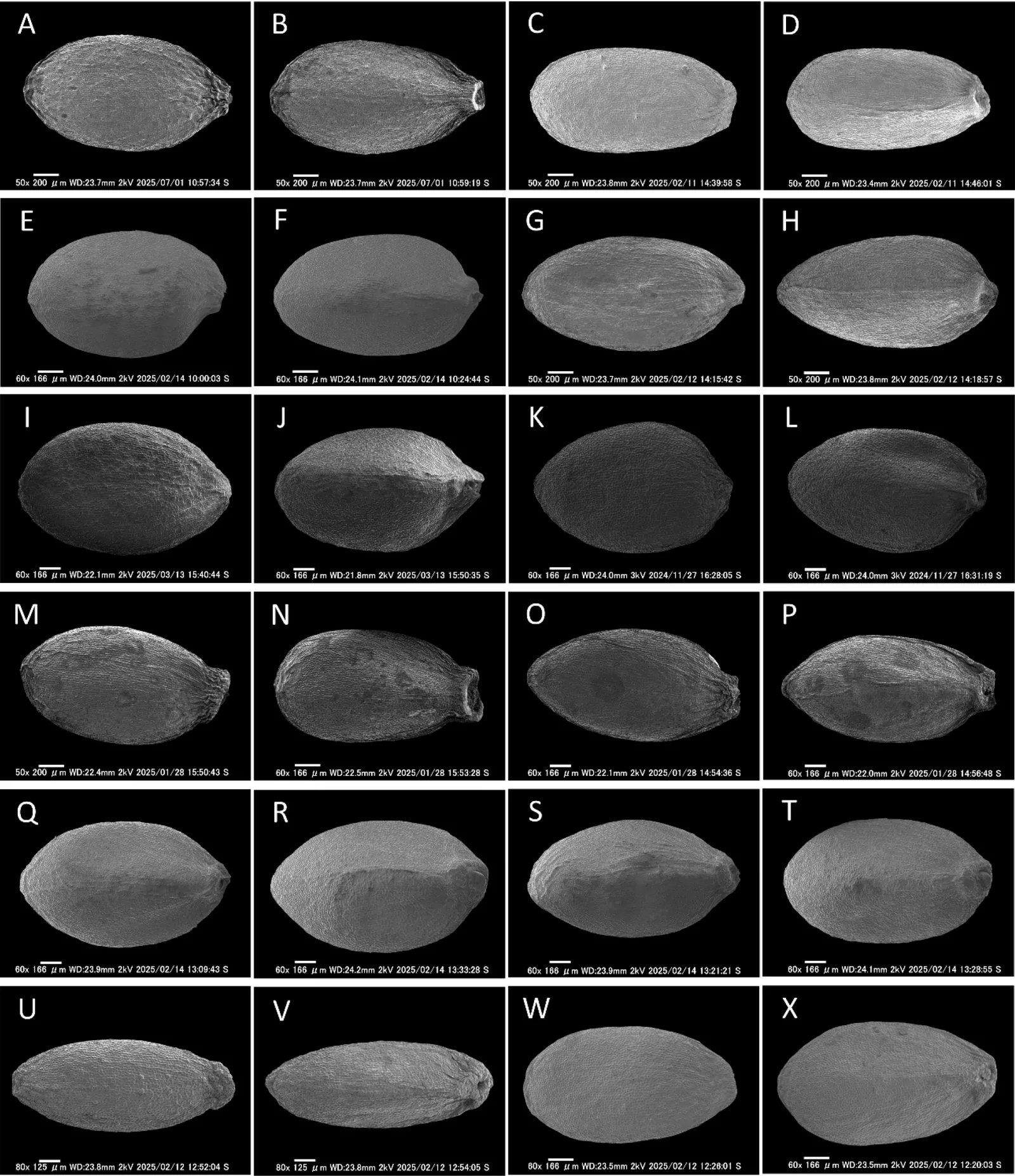
SEM micrographs showing nutlets of *Salvia* sect. *Sobiso*: **A, C, E, G, I, K, M, O, Q, S, U, W**. Dorsal views of nutlets; **B, D, F, H, J, L, N, P, R, T, V, X**. Ventral views of nutlets. Species: **A, B**. *S. akiensis*; **C, D**. *S. isensis*; **E, F**. *S. japonica*; **G, H**. S. lutescens var. lutescens; **I, J**. S. lutescens var. crenata; **K, L**. S. lutescens var. intermedia; **M, N**. S. lutescens var. occidentalis; **O, P**. S. lutescens var. stolonifera; **Q, R**S. omerocalyx var. omerocalyx; **S, T**S. omerocalyx var. prostrata; **U, V**. S. pygmaea var. pygmaea; **W, X**. S. pygmaea var. simplicior. Scale bars: 200 µm (**A, B, C, D, G, H, M**); 166 µm (**E, F, I, J, K, L, N, O, P, Q, R, S, T, W, X**); 125 µm (**U, V**).

**Figure 6.**
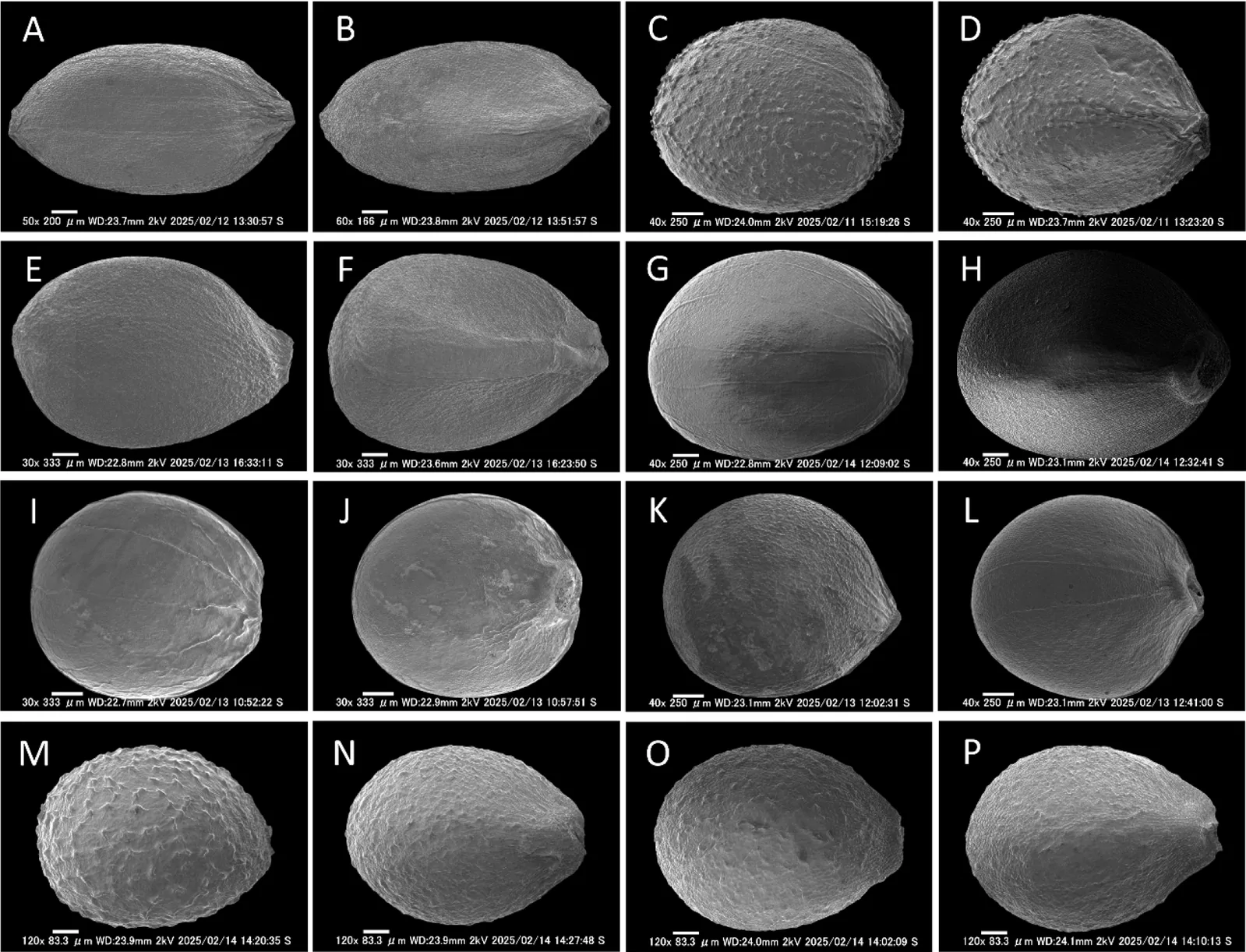
SEM micrographs showing nutlet shape in *Salvia* species: **A, C, E, G, I, K, M, O**. Dorsal views of nutlets; **B, D, F, H, J, L, N, P**. Ventral views of nutlets. Sections: **A, B**. Sect. *Sobiso*; **C, D**. Sect. *Heterosphace*; **E–L**. Sect. *Glutinaria*; **M–P**. Sect. *Notiosphace*. Species: **A, B**. *S. ranzaniana*; **C, D**. *S. lyrata*; **E, F**. *S. koyamae*; **G, H**. *S. nipponica*; **I, J**. S. glabrescens var. glabrescens; **K, L**. S. glabrescens var. repens; **M–P**. *S. plebeia*. Scale bars: 333 µm (**E, F, I, J**); 250 µm (**C, D, G, H, K, L**); 200 µm (**A**); 166 µm (**B**); 83.3 µm (**M, N, O, P**).

**Figure 7.**
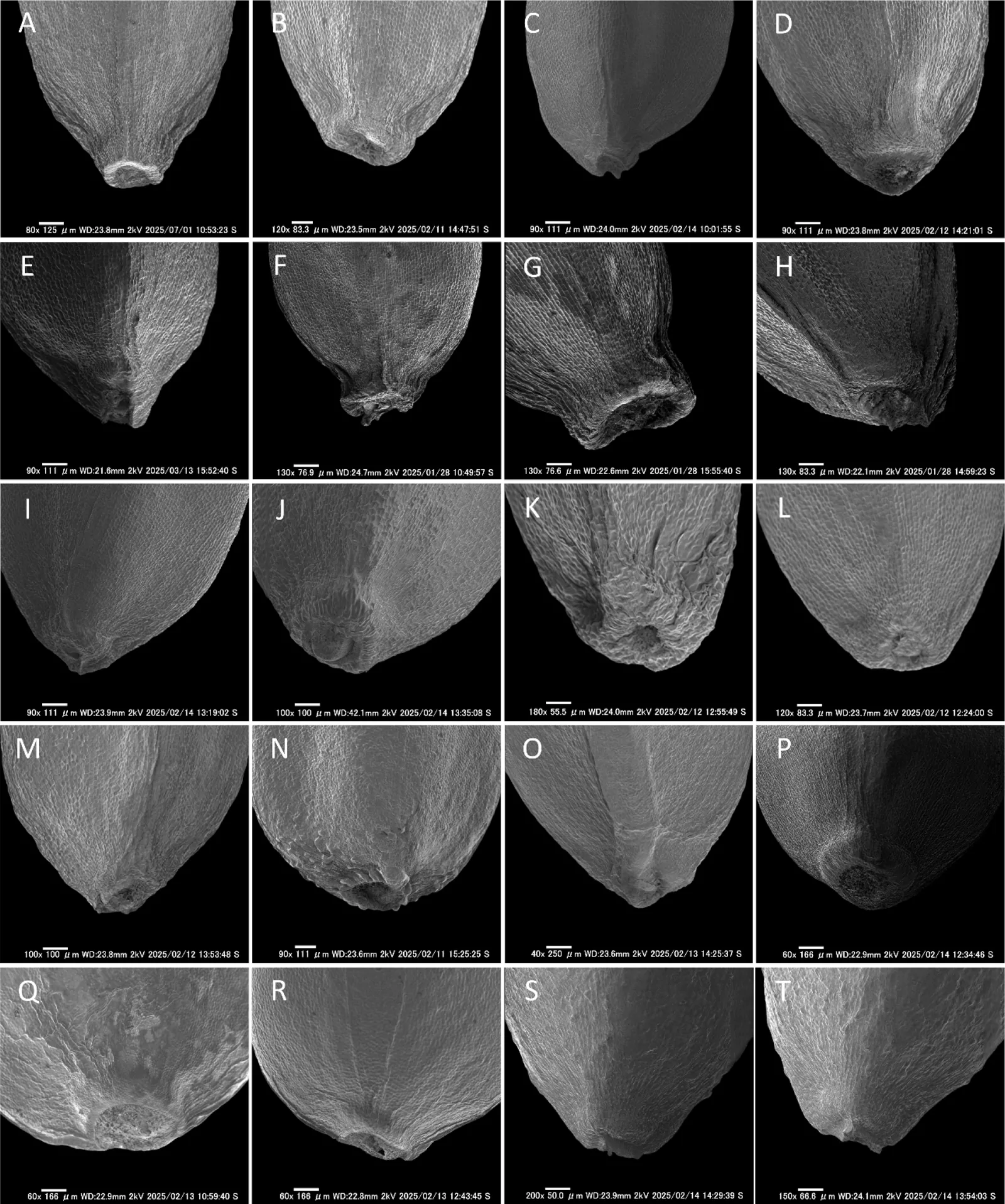
SEM micrographs showing nutlet abscission scars in *Salvia* species: **A**. *S. akiensis*; **B**. *S. isensis*; **C**. *S. japonica*; **D**. S. lutescens var. lutescens; **E**. S. lutescens var. crenata; **F**. S. lutescens var. intermedia; **G**. S. lutescens var. occidentalis; **H**. S. lutescens var. stolonifera; **I**. S. omerocalyx var. omerocalyx; **J**. S. omerocalyx var. prostrata; **K**. S. pygmaea var. pygmaea; **L**. S. pygmaea var. simplicior; **M**. *S. ranzaniana*; **N**. *S. lyrata*; **O**. *S. koyamae*; **P**. *S. nipponica*; **Q**. S. glabrescens var. glabrescens; **R**. S. glabrescens var. repens; **S, T**. *S. plebeia*. Scale bars: 250 µm (**O**); 166 µm (**P, Q, R**); 125 µm (**A**); 111 µm (**C, D, E, I, N**); 100 µm (**J, M**); 83.3 µm (**B, H, L**); 76.9 µm (**F**); 76.6 µm (**G**); 66.6 µm (**T**); 55.5 µm (**K**); 50 µm (**S**).

**Figure 8.**
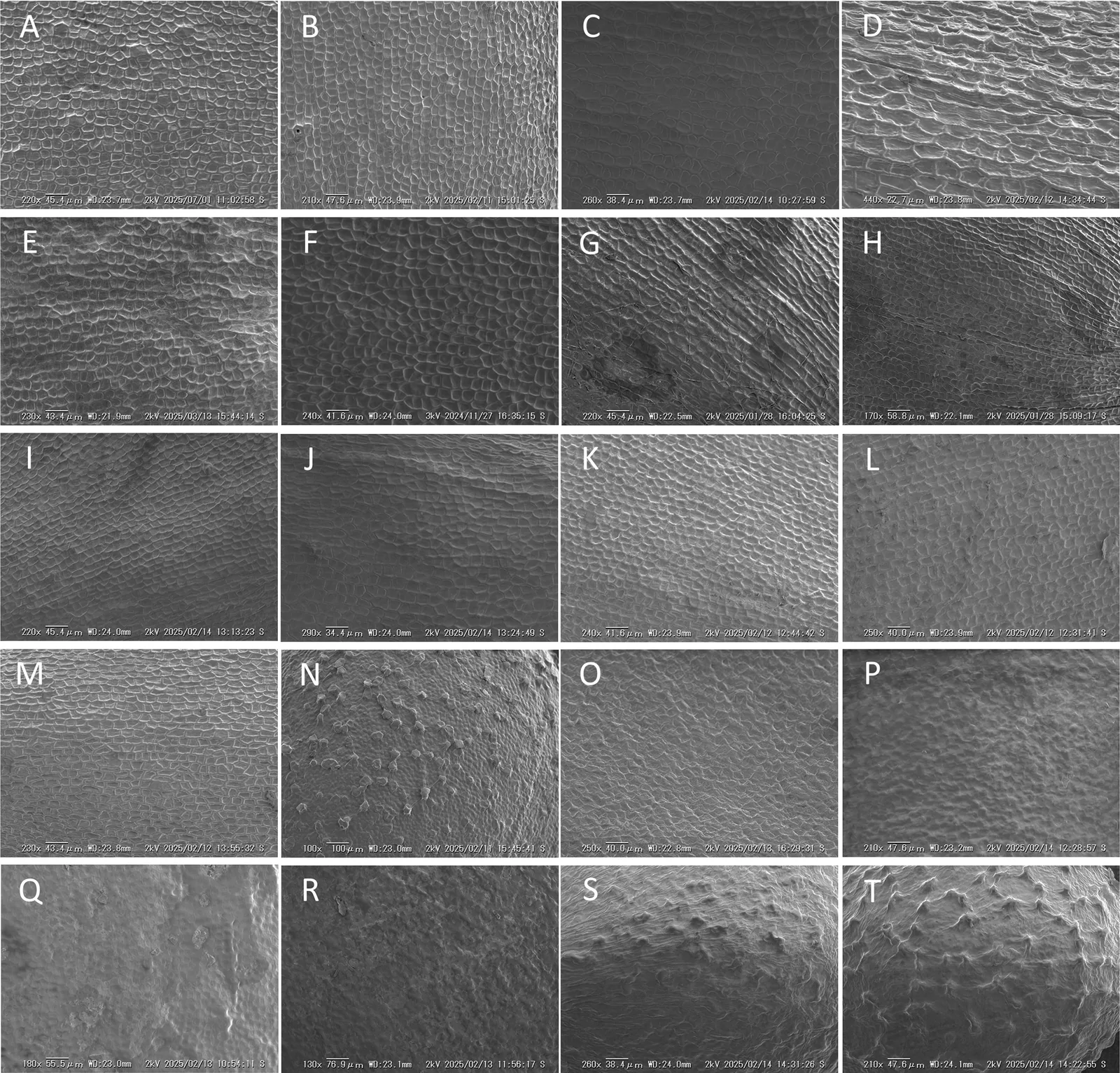
SEM micrographs showing nutlet surface ornamentation at low magnification in *Salvia* species: **A**. *S. akiensis*; **B**. *S. isensis*; **C**. *S. japonica*; **D**. S. lutescens var. lutescens; **E**. S. lutescens var. crenata; **F**. S. lutescens var. intermedia; **G**. S. lutescens var. occidentalis; **H**. S. lutescens var. stolonifera; **I**. S. omerocalyx var. omerocalyx; **J**. S. omerocalyx var. prostrata; **K**. S. pygmaea var. pygmaea; **L**. S. pygmaea var. simplicior; **M**. *S. ranzaniana*; **N**. *S. lyrata*; **O**. *S. koyamae*; **P**. *S. nipponica*; **Q**. S. glabrescens var. glabrescens; **R**. S. glabrescens var. repens; **S, T**. *S. plebeia*. Scale bars: 76.9 µm (**R**); 58.8 µm (**N**); 55.5 µm (**Q**); 47.6 µm (**A, B, P, T**); 45.4 µm (**I**); 43.4 µm (**E, M**); 41.6 µm (**F, K**); 40 µm (**L, O**); 38.4 µm (**S**); 37 µm (**C, J**); 27 µm (**G, H**); 22.7 µm (**D**).

**Figure 9.**
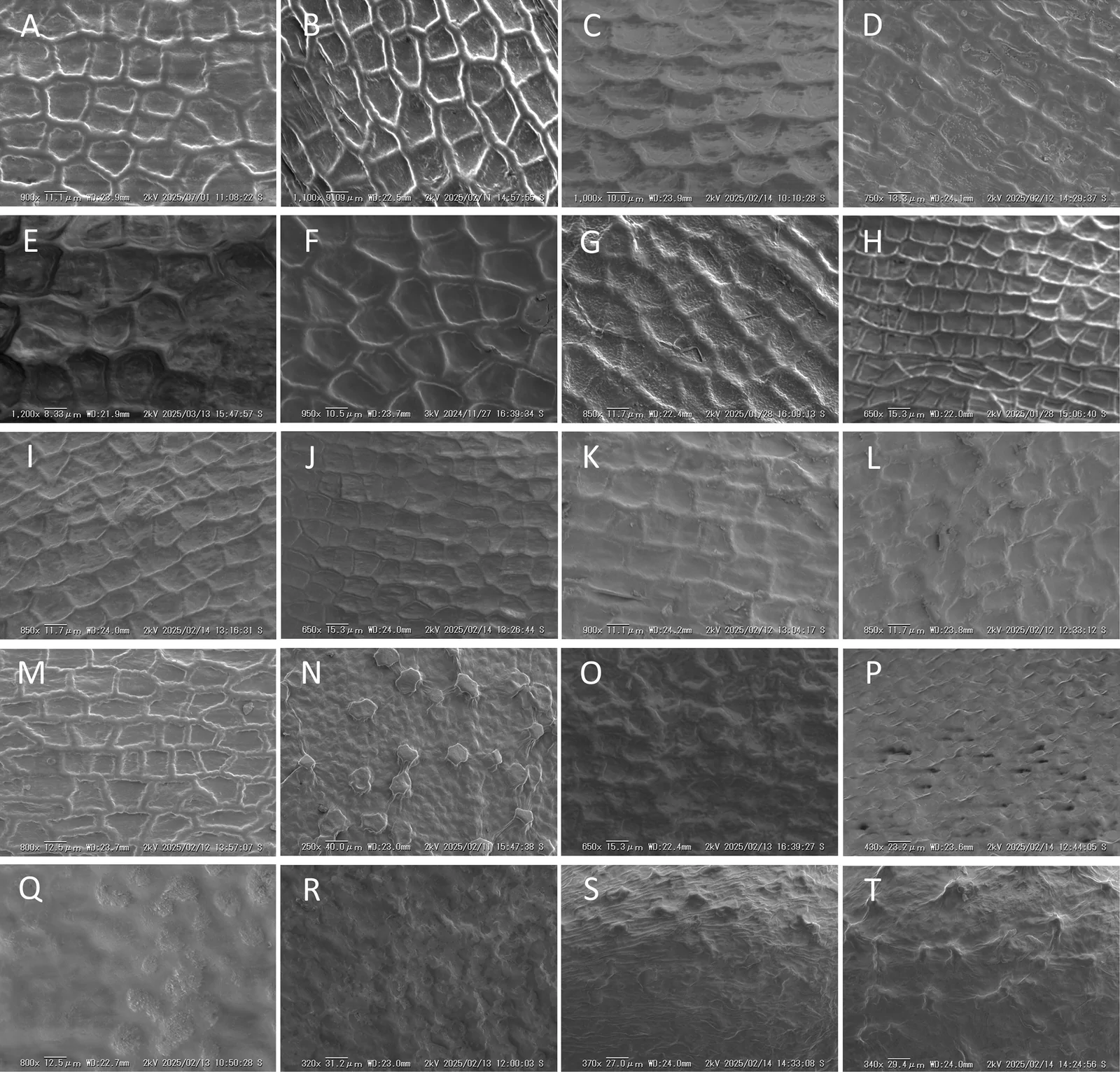
SEM micrographs showing nutlet surface ornamentation at high magnification in *Salvia* species: **A**. *S. akiensis*; **B**. *S. isensis*; **C**. *S. japonica*; **D**. S. lutescens var. lutescens; **E**. S. lutescens var. crenata; **F**. S. lutescens var. intermedia; **G**. S. lutescens var. occidentalis; **H**. S. lutescens var. stolonifera; **I**. S. omerocalyx var. omerocalyx; **J**. S. omerocalyx var. prostrata; **K**. S. pygmaea var. pygmaea; **L**. S. pygmaea var. simplicior; **M**. *S. ranzaniana*; **N**. *S. lyrata*; **O**. *S. koyamae*; **P**. *S. nipponica*; **Q**. S. glabrescens var. glabrescens; **R**. S. glabrescens var. repens; **S, T**. *S. plebeia*. Scale bars: 31.2 µm (**R**); 29.4 µm (**T**); 27 µm (**S**); 23.2 µm (**P**); 15.3 µm (**H, J, O**); 14.2 µm (**N**); 13.3 µm (**D**); 12.5 µm (**M, Q**); 11.7 µm (**G, I, L**); 11.1 µm (**K**); 10.5 µm (**A, F**); 10 µm (**C**); 9.09 µm (**B**); 8.33 µm (**E**).

In the present study, the nutlet morphology of 19 taxa (species and varieties) assigned to the sections *Glutinaria*, *Heterosphace*, *Notiosphace*, and *Sobiso* was examined. These four groups were easily distinguishable based on micromorphological characteristics, particularly nutlet shape, size, color, abscission scar, and surface ornamentation. The diagnostic characters are summarized in Tables 1, 2 and illustrated in Figs 3, 4, 5, 6, 7, 8, 9.

**Table 2.** Nutlet micromorphological (qualitative) characteristics of Japanese *Salvia*.

**Taxon name**	**Nutlet color**	**Nutlet shape**	**Abscission scar shape**	**Nutlet surface ornamentation**	**Nutlet surface cells shape**	**Pattern of anticlinal walls**
***S. akiensis*** A. Takano, Sera & N. Kurosaki	Dull brown	Elliptic	Squarish	Reticulate	Pentagonal to hexagonal	Slightly sinuate, raised
***S. isensis*** Nakai ex H. Hara	Light brown	Narrow elliptic	Triangular	Reticulate	Pentagonal to hexagonal	Slightly sinuate, raised
***S. japonica*** Thunb.	Brown	Ellipsoidal	Irregular	Reticulate	Pentagonal to hexagonal	Slightly sinuate, raised
***S. lutescens*** (Koidz.) Koidz. **var. lutescens**	Golden-brown with longitudinal stripes	Elliptic	Irregular	Reticulate	Pentagonal to hexagonal	Slightly sinuate, raised
**S. lutescens var. crenata** (Makino) Murata	Brown	Elliptic	Irregular	Reticulate	Pentagonal to hexagonal	Slightly sinuate, raised
**S. lutescens var. intermedia** (Makino) Murata	Brown	Elliptic	Irregular	Reticulate	Pentagonal to hexagonal	Slightly sinuate, raised
**S. lutescens var. occidentalis** A. Takano	Dull brown with longitudinal stripes	Ellipsoidal	Orbicular	Reticulate	Pentagonal to hexagonal	Slightly sinuate, raised
**S. lutescens var. stolonifera** G. Nakai	Golden brown	Elliptic	Irregular	Reticulate	Pentagonal to hexagonal	Slightly sinuate, raised
***S. omerocalyx*** Hayata **var. omerocalyx**	Golden brown with longitudinal stripes	Elliptic	Irregular	Reticulate	Pentagonal to hexagonal	Slightly sinuate, raised
**S. omerocalyx var. prostrata** Satake	Brown	Elliptic	Triangular	Reticulate	Pentagonal to hexagonal	Slightly sinuate, raised
***S. pygmaea*** Matsum. **var. pygmaea**	Golden brown with longitudinal stripes	Narrow elliptic	Squarish	Reticulate	Pentagonal to hexagonal	Slightly sinuate, raised
**S. pygmaea var. simplicior** Hatus. ex T. Yamaz.	Dull brown	Elliptic	Triangular	Reticulate	Pentagonal to hexagonal	Slightly sinuate, raised
***S. ranzaniana*** Makino	Dull brown	Elliptic	Irregular	Reticulate	Pentagonal to hexagonal	Slightly sinuate, raised
***S. lyrata*** L.	Blackish brown	Ovoid	Orbicular	Tuberculate	Irregular polygonal	Slightly raised
***S. koyamae*** Makino	Dark brown	Obovoid	Irregular	Colliculate	Irregular	Raised
***S. nipponica*** Miq.	Brown	Ovoid	Orbicular	Colliculate	Irregular	Raised
***S. glabrescens*** Makino **var. glabrescens**	Brown with longitudinal stripes	Spherical	Orbicular	Colliculate	Irregular	Raised
**S. glabrescens var. repens** (Koidz.) S. Kurosaki	Brown with longitudinal stripes	Spherical	Orbicular	Colliculate	Irregular	Raised
***S. plebeia*** R. Br.	Brown	Ovoid	Triangular	Verrucate	Irregular polygonal	Slightly raised

### Nutlet micromorphology of sect. *Sobiso*

Nutlet coloration varied across taxa, including brown (*Salvia japonica*, S. lutescens var. crenata, S. lutescens var. intermedia, and S. omerocalyx var. prostrata), light brown (*S. isensis*), dull brown (*S. akiensis*, S. pygmaea var. pygmaea, and *S. ranzaniana*), dull brown with longitudinal stripes (S. lutescens var. occidentalis), golden brown (S. lutescens var. stolonifera), and golden brown with longitudinal stripes (S. lutescens var. lutescens, S. lutescens var. occidentalis, S. omerocalyx var. omerocalyx, and S. pygmaea var. pygmaea) (Fig. 3). Nutlet length ranged from 1.26 mm in *S. japonica* to 2.10 mm in *S. ranzaniana*, and width ranged from 0.54 mm in S. pygmaea var. pygmaea to 1.22 mm in *S. ranzaniana*. The L/W ratio varied between 1.68 (*S. japonica*) and 2.54 (S. pygmaea var. pygmaea) (Table 1). Nutlet shapes were elliptic in *S. akiensis*, S. lutescens var. lutescens, S. lutescens var. crenata, S. lutescens var. intermedia, S. lutescens var. stolonifera, S. omerocalyx var. omerocalyx, S. omerocalyx var. prostrata, S. pygmaea var. simplicior, and *S. ranzaniana*; narrow-elliptic in *S. isensis* and S. pygmaea var. pygmaea; and ellipsoidal in *S. japonica* and S. lutescens var. occidentalis (Table 2; Figs 3, 5). The length of the abscission scar ranged from 0.025 mm in *S. akiensis* and *S. isensis* to 0.073 mm in S. omerocalyx var. prostrata, and the width ranged from 0.021 mm in *S. akiensis* to 0.066 mm in S. omerocalyx var. prostrata. The L/W ratio ranged from 0.95 (S. pygmaea var. pygmaea) to 1.43 (*S. japonica*). The abscission scar shape was squarish (*S. akiensis* and S. pygmaea var. pygmaea), triangular (*S. isensis*, S. omerocalyx var. prostrata, and S. pygmaea var. simplicior), irregular (*S. japonica*, S. lutescens var. lutescens, S. lutescens var. crenata, S. lutescens var. intermedia, S. lutescens var. stolonifera, S. omerocalyx var. omerocalyx, and *S. ranzaniana*), and orbicular (S. lutescens var. occidentalis) (Fig. 7). The nutlet surface ornamentation exhibited reticulate sculpturing in all studied taxa of *Salvia* sect. *Sobiso* (Fig. 8). Under high-resolution scanning electron microscopy, surface cells ranged from pentagonal to hexagonal, were rarely irregularly polygonal, and were mostly isodiametric, with distinct, slightly sinuate, raised anticlinal walls (Table 2; Fig. 9).

### Nutlet micromorphology of sect. *Glutinaria*

Nutlet coloration varied among taxa: brown in *Salvia nipponica*, dark brown in *S. koyamae*, and brown with longitudinal stripes in *S. glabrescens* and S. glabrescens var. repens (Fig. 4). Nutlet length ranged from 1.95 mm in S. glabrescens var. repens to 3.27 mm in *S. koyamae*, while width ranged from 1.83 mm (S. glabrescens var. repens) to 2.27 mm (*S. koyamae*). The L/W ratio varied from 1.06 in S. glabrescens var. repens to 1.44 in *S. koyamae* (Table 1). Nutlet shapes were obovoid (*S. koyamae*), ovoid (*S. nipponica*), and spherical (S. glabrescens var. glabrescens and S. glabrescens var. repens) (Table 2; Figs 4, 6). The abscission scar length ranged from 0.042 mm (S. glabrescens var. repens) to 0.080 mm (*S. koyamae*), and the width ranged from 0.030 mm (S. glabrescens var. repens) to 0.080 mm (*S. koyamae*). The L/W ratio ranged from 1.00 (*S. koyamae*) to 1.40 (S. glabrescens var. repens). Abscission scar shapes were irregular in *S. koyamae* and orbicular in S. glabrescens var. glabrescens and S. glabrescens var. repens (Fig. 7). The nutlet surface ornamentation exhibited colliculate sculpturing in all studied taxa of *Salvia* sect. *Glutinaria* (Fig. 8). Under high-resolution scanning electron microscopy, the nutlet surface cells appeared irregular, with indistinct, raised anticlinal walls (Table 2; Fig. 9).

### Nutlet micromorphology of sect. *Notiosphace*

Sect. *Notiosphace* was represented in Japan by a single species, *Salvia plebeia*. The nutlets were brown in color and ovoid in shape (Figs 4, 6). The nutlet length was 0.64 mm, and the width was 0.45 mm, resulting in an L/W ratio of 1.42. The abscission scar measured 0.010 mm in both length and width, with an L/W ratio of 1.00 (Table 1). The abscission scar was triangular in shape (Fig. 7). The nutlet surface ornamentation exhibited verrucate sculpturing with numerous small wart-like projections (Fig. 8). Under high-resolution scanning electron microscopy, the nutlet surface was characterized by irregular polygonal cells with slightly raised anticlinal walls (Table 2; Fig. 9).

### Nutlet micromorphology of sect. *Heterosphace*

*Salvia lyrata* was the only species belonging to sect. *Heterosphace* examined in this study. The nutlets were blackish-brown in color and ovoid in shape (Figs 4, 6). The nutlet length was 1.91 mm, with a width of 1.78 mm, resulting in an L/W ratio of 1.07. The abscission scar measured 0.031 mm in length and 0.032 mm in width, with an L/W ratio of approximately 0.96 (Table 1). The abscission scar was orbicular in shape (Fig. 7). The nutlet surface ornamentation exhibited tuberculate sculpturing with prominent knob-like irregular projections (Fig. 8). Under high-resolution scanning electron microscopy, the nutlet surface was characterized by irregular polygonal cells with slightly raised anticlinal walls (Table 2; Fig. 9).

### Nutlet surface sculpturing in Japanese *Salvia*

Based on surface ornamentation, nutlets of the investigated taxa were categorized into four surface sculpturing types across the following sections:

Type I. **Reticulate**: Net-like pattern formed by distinct pentagonal to hexagonal cells (sect. *Sobiso*).

Type II. **Tuberculate**: Surface bearing prominent knobs like irregular projections (sect. *Heterosphace*).

Type III. **Colliculate**: Surface low, rounded hill-like elevations (sect. *Glutinaria*).

Type IV. **Verrucate**: Surface covered with numerous, small wart-like projections (sect. *Notiosphace*).

### Multivariate analysis of nutlet micromorphology

**Figure 10.**
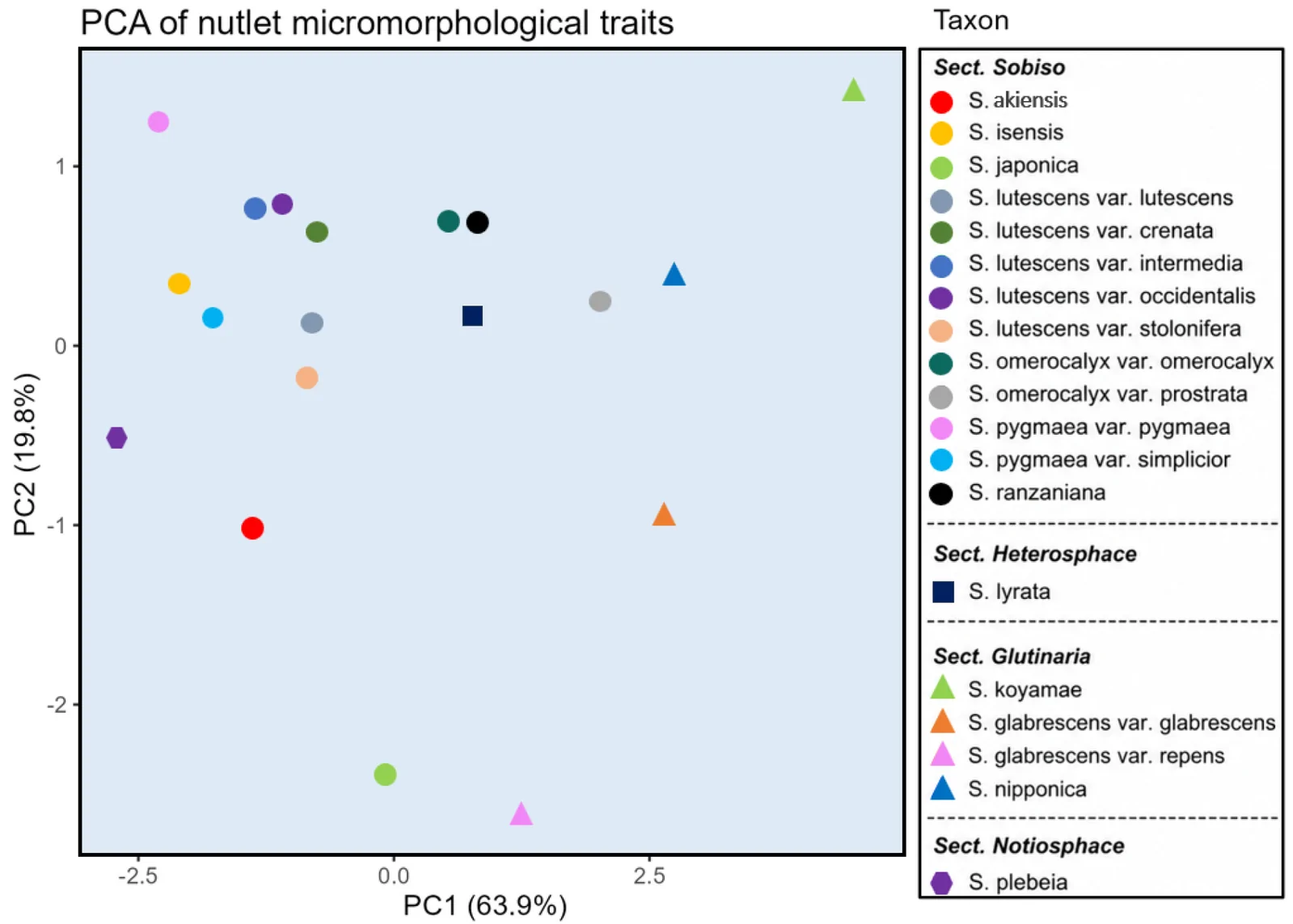
PCA score plot of quantitative nutlet characters based on mean values for 19 taxa of Japanese *Salvia*. Each point represents one taxon mean. Different symbols indicate sections, and different colors indicate individual taxa.

PCA was performed using the mean values of six quantitative nutlet characters (nutlet length, nutlet width, nutlet L/W ratio, abscission scar length, abscission scar width, and abscission scar L/W ratio) for the 19 investigated taxa (species and varieties). Although measurements were obtained from 84 specimens, the PCA was conducted at the taxon level because complete specimen-level data for all six variables were not available for every specimen. Thus, each point in the PCA represents one taxon mean (Fig. 10). PC1 and PC2 together explained 83.7% of the total variation, with PC1 accounting for 63.9% and PC2 for 19.8%. PC1 showed similar positive loadings for nutlet length (0.471) and abscission scar width (0.467), with a weaker negative loading for the nutlet L/W ratio (−0.353). Therefore, PC1 primarily represents overall variation associated with nutlet and abscission scar size. *Salvia* species with the highest PC1 scores (*S. koyamae*, S. glabrescens var. glabrescens, and *S. nipponica*) grouped toward the positive side of PC1, reflecting their relatively larger nutlet and abscission scar dimensions. In contrast, the lowest PC1 scores were found in *S. akiensis*, *S. isensis*, S. pygmaea var. pygmaea, S. pygmaea var. simplicior, *S. plebeia*, and *S. lutescens* varieties, all of which have the smallest nutlet and scar dimensions. PC2 was dominated by the negative loading for the abscission scar L/W ratio (−0.841), with a secondary positive contribution from the nutlet L/W ratio (0.414). PC2 was strongly influenced by the L/W ratio, separating species primarily by shape rather than size. The most negative PC2 scores belonged to *S. japonica* and varieties of *S. glabrescens*, which have the highest abscission scar L/W ratios. The most positive PC2 score belonged to S. pygmaea var. pygmaea, which has the highest nutlet L/W ratio and one of the lowest abscission scar L/W ratios (Table 1). Overall, the PCA based on taxon means demonstrates that nutlet and abscission scar size contributed strongly to the observed variation among taxa, while proportional characters provided additional differentiation among the investigated taxa (Fig. 10).

## Discussion

Even with a limited number of taxa, the present results demonstrate that nutlet morphology can contribute significantly to the existing infrageneric classification of *Salvia*. This study provides the first comprehensive account of nutlet morphology in Japanese *Salvia*, representing four sections: *Glutinaria*, *Heterosphace*, *Notiosphace*, and *Sobiso*. Significant differences were observed across all examined nutlet characters, establishing a robust morphological framework for evaluating both interspecific and intraspecific variation within Japanese *Salvia*. The findings of the present study and previous literature show that nutlet morphological characters are useful not only for species identification but also for distinguishing sections and subgenera across the genus (Marín et al. 1996; Moon et al. 2009; Özkan et al. 2009; Kahraman et al. 2010a, 2010b; Kahraman 2011; Büyükkartal et al. 2011; Mousavi et al. 2013; Celep et al. 2014; Salimpour et al. 2014; Polat et al. 2015; Li et al. 2016; Alimpić et al. 2021; Kılıç and Kılıç 2021; Celep et al. 2022).

Mature nutlets of *Salvia* usually vary from light brown-yellow and brown to blackish-brown, often with darker stripes or a mottled appearance across a remarkably broad taxonomic range within the genus, including sections *Aethiopis*, *Angulatae*, *Calosphace*, *Drymosphace*, *Hemisphace*, *Holochilus*, *Horminum*, *Hymenosphace*, *Plethiosphace*, and *Salvia*, as well as subgenera *Salvia*, *Sclarea*, and *Heterosphace* (Özkan et al. 2009; Moon et al. 2009; Kahraman et al. 2010a; Kahraman 2011; Ifrim 2012; Salimpour et al. 2014; Ananthalakshmi 2020; Celep et al. 2022). In addition, specifically pale brown nutlets have been reported in sect. *Plethiosphace* and sect. *Salvia* (Büyükkartal et al. 2011; Alimpić et al. 2021), and blackish to brown-yellowish nutlets have been reported within sect. *Salvia* (Kılıç and Kılıç 2021). The present observations align with this consensus, indicating that brown coloration with darker markings is a broadly conserved feature across Old World *Salvia* rather than a section-specific diagnostic character, and its taxonomic importance mainly relies on fine-scale variation (marking patterns) rather than nutlet color patterns.

Nutlet shape has long been regarded as a taxonomically informative character in *Salvia* (Oran 1996; Kahraman et al. 2010a). The diversity reported in earlier work includes elliptic, widely elliptic, ovate, lance-ovate, oblong, obovate, ovoid, suborbicular, and trigonous forms across *Salvia* (Harley et al. 2004; Moon et al. 2009; Kahraman et al. 2010b; Büyükkartal et al. 2011; Mousavi et al. 2013; Celep et al. 2014; Polat et al. 2015; Li et al. 2016; Alimpić et al. 2021). These observations corroborate the present study. However, the present findings diverge from the widely reported pattern of assessing circular (spheroidal) and prolate shapes primarily via the L/W ratio (Özkan et al. 2009; Kahraman et al. 2010a; Kılıç and Kılıç 2021), suggesting that nutlet shape descriptions in Japanese *Salvia* contradict this trend. In the studied taxa, the most common nutlet shape is elliptic, which has also been reported in some members of sections *Aethiopis*, *Holochilus*, *Hemisphace*, *Horminum*, *Hymenosphace*, *Plethiosphace*, and *Salvia* (Salimpour et al. 2014; Ananthalakshmi 2020). According to Celep et al. (2022), the basic nutlet shapes reported across subgenera *Salvia*, *Sclarea*, and *Heterosphace* include elliptic, widely elliptic, circular, ovate, and obovate forms. Notably, the long-ovoid nutlet shape observed in *S. plebeia* corresponds precisely with descriptions of this species by Sales et al. (2010) and Li et al. (2016), providing independent cross-study confirmation.

Nutlet size has repeatedly proven to be a diagnostic character in *Salvia* (Moon et al. 2009; Kahraman et al. 2010b). Nutlet length and width ranges vary among the infrageneric groups of *Salvia*: 1.6–3.5 mm in length and 1.0–2.9 mm in width across taxa of sections *Plethiosphace*, *Salvia*, *Hemisphace*, and *Hymenosphace* (Özkan et al. 2009); 1.53–2.64 mm in length and 1.00–2.39 mm in width across taxa of sections *Aethiopis*, *Holochilus*, *Hemisphace*, *Hymenosphace*, and *Plethiosphace* (Salimpour et al. 2014); 2–3.5 mm in length and 1.5–2.5 mm in width across sections *Salvia*, *Plethiosphace*, *Aethiopis*, and *Horminum* (Ananthalakshmi 2020); 3.02–6.47 mm in length and 1.78–5.10 mm in width in taxa of sect. *Salvia* (Kılıç and Kılıç 2021); and 1.61–8.44 mm in length and 1.02–4.20 mm in width, with L/W ratios of 1.02–3.06, across subgenera *Heterosphace*, *Salvia*, and *Sclarea* (Celep et al. 2022). These measurements are comparable to the present findings. Nutlet size ranges of 1.5–2.5 × 1.0–2.0 mm in sect. *Plethiosphace* and 2.2–2.5 × 1.3–1.5 mm in sect. *Hemisphace* (Büyükkartal et al. 2011) are compatible with those observed in the present study. The smallest nutlets recorded in *S. plebeia* (0.45–0.60 × 0.38–0.40 mm) correspond with the measurements reported by Sales et al. (2010) and Li et al. (2016) for the same species, suggesting that nutlet size may represent a relatively consistent character at the species level.

Abscission scar morphology has been considered systematically useful in *Salvia* (Harley et al. 2004; Moon et al. 2009; Özkan et al. 2009). Several studies have reported abscission scar position, shape, and diameter as descriptive characters across different sections of the genus: triangular scars in sections *Aethiopis* and *Plethiosphace*; orbicular scars in sections *Aethiopis*, *Hemisphace*, *Holochilus*, *Hymenosphace*, and *Plethiosphace*; round scars in sections *Salvia* and *Hemisphace*; triangular scars in sections *Plethiosphace* and *Eusphace*; and irregular scars in sections *Holwaya* and *Drymosphace* (Kahraman 2011; Büyükkartal et al. 2011; Ifrim 2012; Salimpour et al. 2014; Alimpić et al. 2021). Furthermore, an areolate to round abscission scar has been reported in subg. *Salvia* and a triangular scar in subg. *Sclarea* (Celep et al. 2022). Additionally, Li et al. (2016) described a convex-to-round abscission scar in *S. plebeia*, whereas a triangular scar was observed in the same species in the present study. Abscission scar diameter has been reported to range from 0.50–1.28 mm, with L/W ratios of 1.01–1.88 among taxa of sect. *Hymenosphace* (Kahraman et al. 2010b; Kahraman 2011), and scar diameters of 0.41–0.83 mm have been reported among species of sect. *Salvia* (Büyükkartal et al. 2011). These findings lend additional support to the present results. However, these studies suggest that the abscission scar is a consistent, apomorphic trait that carries taxonomic information at the species level, extending to the sectional and even subgeneric levels within *Salvia*.

Nutlet surface sculpturing has been utilized as a key diagnostic character for species identification at the sectional level within *Salvia* (Kahraman et al. 2010a; Kahraman 2011; Kılıç and Kılıç 2021). The present results showed some inconsistencies with the findings of previous studies (Moon et al. 2009; Özkan et al. 2009; Kahraman et al. 2010b; Mousavi et al. 2013; Salimpour et al. 2014). In contrast, Kılıç and Kılıç (2021) reported a colliculate pattern in sect. *Salvia*, which is consistent with the present findings. Similarly, Celep et al. (2022) described subgenus-specific ornamentation patterns: colliculate-verrucate with small knobs in subg. *Heterosphace*, colliculate with rounded knobs in subg. *Sclarea*, and a more complex colliculate-reticulate or verrucate pattern with pentagonal-hexangular, sinuate-walled cells in subg. *Salvia*, which broadly correspond with the ornamentation patterns observed in the present study. At the species level, the rough, non-reticulate or uneven nutlet surface of *S. plebeia* agrees with Li et al. (2016). The tuberculate ornamentation with small knob-like projections observed in *S. lyrata* is consistent with the original description by Marín et al. (1996). These comparisons demonstrate that nutlet ornamentation is a relatively stable character with considerable taxonomic importance for species identification and infrageneric classification in *Salvia*. The present results extend this pattern to the previously uncharacterized Japanese lineage.

The multivariate analysis of nutlet micromorphological traits provides additional information on patterns of variation among the investigated Japanese *Salvia* taxa. Because the PCA was based on taxon means, these patterns should be regarded as comparative evidence among taxa rather than as direct evidence of within-taxon morphological variation or taxonomic distinctness. PC1 was primarily associated with size-related traits, with nutlet and abscission scar dimensions contributing strongly to the separation of taxon means, making overall nutlet/scar size the single largest source of morphological differentiation. This size axis separates large-nutlet taxa (*Salvia koyamae*, S. glabrescens var. glabrescens, and *S. nipponica*) from small-nutlet taxa (*Salvia akiensis*, *S. isensis*, S. pygmaea var. pygmaea, S. pygmaea var. simplicior, *S. plebeia*, and *S. lutescens* varieties). PC2 was mainly associated with proportional characters, particularly the abscission scar and nutlet L/W ratios, and captured shape independently of size. It distinguished S. glabrescens var. glabrescens, S. glabrescens var. repens, and *S. japonica* by their unusually elongate scars, while S. pygmaea var. pygmaea, despite differing greatly in size, was characterized by narrowly elongate nutlets with proportionally squarish-rounded scars. This shows that size and shape vary independently among the studied taxa, and taxa can resemble each other on one axis while differing on the other. The close grouping of the *S. lutescens* varieties with *S. isensis* and S. pygmaea var. simplicior along both axes supports treating them as a morphologically cohesive complex, whereas the strong isolation of *S. koyamae* on both axes reinforces its status as a well-differentiated taxon.

## Conclusion

The present study demonstrates that nutlet morphology, particularly surface ornamentation, exhibits a high degree of infrageneric stability among Japanese *Salvia*. Four distinct types of surface ornamentation were recognized, corresponding closely with the four investigated sections: reticulate in sect. *Sobiso*, colliculate in sect. *Glutinaria*, verrucate in sect. *Notiosphace*, and tuberculate in sect. *Heterosphace*. The consistency of these ornamentation patterns, together with other nutlet morphological characters, provides additional support for the existing sectional circumscription of Japanese *Salvia* and is congruent with the recently proposed infrageneric classification (Hu et al. 2018). In contrast, some nutlet characters, such as nutlet shape, size, and abscission scar morphology, were more informative for species identification than for supporting the current sectional classification of Japanese *Salvia*.

## Appendix 1

**Table A1.** Voucher information for *Salvia* taxa studied for nutlet micromorphology. (*: Taxa endemic to Japan).

**Taxon name**	**Locality and voucher information**
***S. akiensis*** * A. Takano, Sera & N. Kurosaki	**Sect. *Sobiso* (Raf.) G.X. Hu, A. Takano & B.T. Drew**
	Honshu, Pref. Shimane, Gotsu-shi, Mastukawa-cho, Yakami, alt. 40 m, 24 May 2012, Masahiro Sakoda 9150 (C1-244507 HYO);
	Honshu, Pref. Hiroshima, Yamagata-gun, Akiota-cho, Okutakiyama Gorge, Togouchi, Shimoyama, 34°24'21"N, 132°10'24"E, alt. 430 m, 13 June 2008, Tetsuya Sera s.n. (C1-244506 HYO).
***S. isensis*** * Nakai ex H. Hara	Honshu, Pref. Mie, Ise, Mt. Asama-yama, 30 August 1986, Tomiki Kobayashi 06430 (C2-012147 HYO);
	Honshu (Suga-shima Island), Pref. Mie, Toba-shi, Oyama, 34°29'28"N–34°29'35"N, 136°53'31"E–136°53'42"E, alt. 150–200 m, 15 September 2017, Shinji Fujii s.n. (17551 KYO).
***S. japonica*** Thunb.	Honshu, Pref. Hyogo, Kobe-shi, Kita-ku, North slope of Mt. Rokko-san, en route from Gokuraku-chaya to Arima-cho, alt. 400–850 m, 5 September 1982, T. Shimizu & N. Kurosaki 45 (C2-012292 HYO);
	Honshu, Pref. Hyogo, Kobe-shi, Kita-ku, Yamada-cho, en route from Aina to Sakamoto, alt. 200–300 m, 24 October 1976, N. Kurosaki 8032 (C2-012283 HYO);
	Honshu, Pref. Hyogo, Kobe-shi, Kita-ku, along Ashitani-gawa River, at northern foot of Mt. Chigogabaka-yama, alt. 200–300 m, 11 October 1981, N. Fukuoka 11296 (C2-012296 HYO);
	Honshu, Pref. Hyogo, en route from Hasu (Takarazuka-shi) to Dojo (Kobe-shi) along Sengari-suigenchi, alt. 150–300 m, 16 November, 1975, N. Kurosaki 7089 (C2-012300 HYO);
	Honshu, Pref. Hyogo, Mihara-gun, Mihara-cho, Amano, Umamawari, around Nariai-ji, 34°16'00"N, 134°49'00"E, alt. 100–160 m, 11 December 1992, N. Fukuoka & S. Miyake 5314 (C2-012262 HYO);
	Shikoku (Shodo-shima Island), Pref. Kagawa, Shozu-gun, Tonosho-cho, Myokenzaki, sea side of Myoken-Point, alt. ca. 80 m, 6 August 1985, Miyoshi Furuse s.n. (53410 KYO).
***S. lutescens*** (Koidz.) Koidz. **var. lutescens** *	Honshu, Pref. Mie, Ichishi-gun, Ichishi-mura, near Yazu Pass, Yazu-no-osugi, 25 July 1980, K. Seto 26350 (C2-012696 HYO);
	Honshu, Pref. Mie-ken, Kameyama-shi, Seki-cho, Sakashita, 11 July 2006, S. Tsugaru & T. Sawada 34122 (C2-012697 HYO).
**S. lutescens var. crenata** * (Makino) Murata	Honshu, Pref. Gifu, Ono-gun, Asahi-mura, the valley of Sokubo-dani, alt. 1100–1300 m, 23 July 1974, H. Takahashi 2131 (C2-012687 HYO);
	Honshu, Pref. Gifu, Mugi-gun, Itadori-mura, the ravine of Unnomizo-dani, alt. ca. 500 m, 27 June 1997, H. Takahashi 17194 (C2-012688 HYO);
	Honshu, Pref. Gifu, Mugi-gun, Itadori-mura, the ravine of Unnomizo-dani, alt. ca. 500 m, 30 July 1995, H. Takahashi 16215 (C2-012689 HYO);
	Honshu, Pref. Gifu, Yamagata-gun, Kitayama-mura, Kanzaki, 1 June 1937, G. Koidzumi s.n. (C2-012690 HYO);
	Honshu, Pref. Nagano, Shimoina-gun, Oshika-mura, Mt. Shiomi-dake, Hontani & Nutami-dake, alt. 1750–1800 m, 9 August 2009, Satoru Takeshige 01 (090809 KYO);
	Pref. Niigata, Minamiuonuma-gun, Yuzawa-cho, Oaza Mikuni, Mikuni Pass, 36°46'08"N, 138°48'58"E, alt. 1092 m, 27 July 2023, A. Takano 230726-8 (C1-284420 HYO);
	Honshu, Pref. Kanagawa, Yamakita-cho, Yushin Valley, 35°26'11"N, 139°05'59"E, alt. 550 m, 4 July 2007, S. Fuse & Y. Chikawa 4556 (C1-229808 HYO);
	Honshu, Pref. Gunma, Takasaki-shi, Harunako-machi, Mt. Haruna-san, 36°28'44"N, 138°52'30"E, alt. 1128 m, 26 July, 2023, A. Takano 230726-3 (C1-284415 HYO);
	Honshu, Pref. Nagano, Chiisagata-gun, Nagawa-machi, Daimon, Echovalley snow resort, 36°07'N, 138°12'E, alt. 1500–1600 m, 24 July 2014, Tomiki Kobayashi 51846 (C1-253856 HYO);
	Honshu, Pref. Fukui, Katsuyama-shi, Yoshino, Mt. Hoonji-san, 36°3'44"N, 136°35'23"E, alt. 1130 m, 2 July 2015, A. Takano 150702-1a (C1-254735 HYO);
	Pref. Ishikawa, Mt. Haku-san, Sannomine, 9 August 1968, M. Hashimoto 7707 (C1-069478 HYO);
	Honshu, Pref. Kanagawa, Ashigarashimo-gun, Sengokuhara, Kintoki Shrine, 35°16'47"N, 139°0'11"E, alt. 720 m, 6 August 2014, A. Takano 140806-2 (C1-248549 HYO).
**S. lutescens var. intermedia** * (Makino) Murata	Honshu, Pref. Gunma, Mt. Shibutsu-san, 11 August 1977, s.coll., s.n. (C2-012676 HYO);
	Honshu, Pref. Shizuoka, Tatsuyama-mura, Shirakura, alt. 400 m, 22 August 2002, Michito Ohta B-2002-005 (C2-012686 HYO);
	Honshu, Pref. Gunma, Oze, Mt. Hiuchi-dake, 2 August 1950, Yoshimasa Matsumura s.n. (C2-012692 HYO);
	Honshu, Pref. Shizuoka, Tagata-gun, Amagiyugashima-cho, Kayano, vicinity of the Joren fall, 4 August 1970, M. Ichikawa, T. Fujimura & F. Konta 1386 (C2-012700 HYO).
**S. lutescens var. occidentalis** * A. Takano	Honshu, Pref. Nara, Yamato Province, Yoshin-gun, Mt. Sanjogatake, s.d. Y. Araki 10898 (C2-012704 HYO);
	Honshu, Pref. Nara, Yoshino-gun, Kamikitayama-mura, between Mt. Wasamata-yama & Mt. Daihugen-dake in the Mts. Omine-san, alt. 1400–1760 m, 19 August 1984, G. Murata 01 (45421 KYO);
	Honshu, Pref. Nara (Prov. Yamato), 13 July 1922, G. Koizumi s.n. (130722 KYO);
	Honshu, Pref. Nara, Yoshino-gun, higher elevation of Mt. Daifugen-dake from Mt. Wasamata-yama in Mt. Omine-san, alt. 1500 m, 12 August 1977, H. Koyama & M. Hotta s.n. (5410 KYO);
	Honshu, Pref. Shiga, en route Tochu to Ikadate, alt. ca. 300 m, 2 August 1980, M. Umebayashi s.n. (737 KYO);
	Honshu, Pref. Shizuoka, Kamitani, Fuji-shi, 35°11'43"N, 138°46'19"E, alt. 370 m, 15 July 2019, A. Takano 20190715-1 (C1-271465 HYO);
	Honshu, Pref. Nara, Mt. Ofugen-dake, 11 August 1967, S. Hosomi 5104 (C2-012706 HYO);
	Honshu, Pref. Hyogo, Sanda-shi, Moshi, 35°01'00"N, 135°14'00"E, alt. ca. 460 m, 19 August 1995, A. Takahashi 3119 (C2-012711 HYO);
	Honshu, Pref. Hyogo, Sanda-shi, Eitakuji, 10 August 1977, H. Hosomi 17692-1 (C1-004169 HYO);
	Honshu, Pref. Hyogo, Sanda-shi, Mohshi-oike, 35°01'00"N, 135°12'00"E, alt. 450 m, 19 August 1994, A. Takahashi 2567 (C1-077715 HYO);
	Shikoku, Pref. Kochi, Tosa-gun, Tosa-cho, Mt. Mitsuji-yama, 33°41'00"N, 133°30'00"E, alt. 1000 m, 14 July 2014, A. Takano, A. Sakamoto & Y. Tanabe 140729-8 (C1-248544 HYO);
	Honshu, Pref. Hyogo, Sanda-shi, Eitakuji, 19 September 1936, H. Hosomi 2692 (C1-018755 HYO);
	Honshu, Pref. Nara, Yoshino-gun, Kamikitayama-mura, en route from Mt. Wasamata hut to Mt. Nihon-dake through the cliff Sho-no-Iwaya, alt. ca. 1200–1500 m, 5 August 1978, M. Okamoto 1966 (C2-012708 HYO).
**S. lutescens var. stolonifera** * G. Nakai	Honshu, Pref. Aichi, Mt. Dando-san, 18 June 1953, G. Murata s.n. (180653 KYO);
	Honshu, Pref. Tochigi, Mu Changhui, Ohira Pass, 21 July 1974, K. Okuhara s.n. (210774 KYO);
	Honshu, Pref. Aichi, Mt. Dando-san, 1 September 1951, Kiichi Torii s.n. (010951 KYO);
	Honshu, Pref. Aichi, Mt. Dando-san, 21 June 1933, Suzuki Kugijiro s.n. (210633 KYO).
***S. omerocalyx*** Hayata **var. omerocalyx** *	Honshu, Pref. Hyogo, Mikata-gun, Kami-cho, Mt. Mikawa-yama, 16 June 1968, S. Hosomi s.n. (C1-018584 HYO);
	Honshu, Pref. Hyogo, Kinosaki-gun, Hidaka-cho, Inanba, 35°30'56"N, 134°39'2"E, alt. ca. 570 m, 10 June 2001, A. Takahashi & S. Fuse 4989 (C1-233707 HYO);
	Honshu, Pref. Hyogo, Kinosaki-gun, Hidaka-cho, Inanba, 35°28'N, 134°42'E, alt. ca. 400 m, 24 June 2000, S. Nishida, S. Miyake, S. Nakao & T. Nishida 1580 (C1-150309 HYO);
	Honshu, Pref. Hyogo, Toyooka-shi, Takeno-cho, Suwa Shrine, 35°39'26"N, 134°45'37"E, alt. 50 m, 27 May 2006, A. Takano & S. Fuse 060527-13 (C1-220283 HYO).
**S. omerocalyx var. prostrata** * Satake	Honshu, Pref. Fukui, Nanjo-gun, Minamiechizen-cho, Hirono, 35°43'30"N, 136°17'6"E, alt. 400 m, 4 July 2015, A. Takano 150704-14 (C1-254775 HYO).
***S. pygmaea*** Matsum. **var. pygmaea** *	Kyushu (Iriomote-jima Island), Pref. Okinawa, stream near the Kanpire Waterfall, alt. ca. 460 m, 23 April 2004, H. Setoguchi JP2446 (00068797 KYO);
	Kyushu (Iriomote-jima Island), Pref. Okinawa, along a Nakama-gawa River, near otochi, 23 October 1996, K. Iwatsuki et al. 710 (00068796 KYO);
	Kyushu (Okinawa Island), Pref. Okinawa, Kunigami-gun, Kunigami-son, Aha, Tanagagumui, 26°43'20"N, 128°17'20"E, 9 July 2003, Akiyo Naiki & Shinji Fujii 5465 (00068807 KYO).
**S. pygmaea var. simplicior** * Hatus. ex T.Yamaz.	Kyushu (Tokunoshima Island), Pref. Kagoshima, Oshima-gun, Mt. Inokawa-dake: along the trail from the TV tower to the summit, 27°45'00"N–27°46'00"N, 128°58'00"E–129°00'00"E, alt. 430–645 m, 10 October 1986, M. Tamura et al. 52 (00068833 KYO).
***S. ranzaniana*** * Makino	Shikoku, Pref. Kochi, Aki-shi, Becchaku, near Komase Tunnel upstream of Ioki River, 23 March 2007, Tessin Yamawaki 253 (107187 KYO);
	Honshu, Pref. Mie, Kitamuro-gun, Kiinagashima-cho, Mito, Sando River, a branch of Akaba River, 34°11'50"N–34°12'05"N, 136°15'30"E–136°16'15"E, alt. 50-70 m, 8 May, 2005, Shinji Fujii s.n. (10698 KYO);
	Honshu, Pref. Mie, Kitamuro-gun, Kihoku-cho, Miyama-ku, Nakasato, 34°08'25"N, 136°13'15"E, alt. 10-30 m, 11 June 2012, Shinji Fujii & K. Yamamoto s.n. (15509 KYO);
	Honshu, Pref. Nara, Shimokitayama-mura, Ikemine, near Myojin Pond, 34°1'56"N, 135°57'25"E, alt. 400 m, 12 May 2002, Kazuhiro Kawabata s.n. (4782 KYO).
***S. lyrata*** L.	**Sect. *Heterosphace* Benth.**
	Honshu (Nii-jima Island), Tokyo Metropolis, road to Mt. Miyatsuka-yama, 29 April 2023, H. Hayakawa et al. HH948 (Mus. Nat. Env. Hist., Shizuoka);
	Honshu (Nii-jima Island), Tokyo Metropolis, road to Mt. Miyatsuka-yama, 5 June 2022, T. Yamamoto HH697 (Mus. Nat. Env. Hist., Shizuoka);
	Honshu, Pref. Hyogo, Kobe-shi, Chou-ku, South of Sannomiya 34°41'15.3"N, 135°11'35.9"E, alt. 29 m, 3 September 2025, Amjad Khan s.n. (C1-292136 HYO).
***S. koyamae*** * Makino	**Sect. *Glutinaria* (Raf.) G.X. Hu, C.L. Xiang & B.T. Drew**
	Honshu, Pref. Nagano, Minamisaku-gun, Kitamaki-mura, side of Matsubara-Lake, 25 September 1958, Miyoshi Furuse s.n. (C1-119444 HYO).
***S. glabrescens*** Makino **var. glabrescens** *	Honshu, Pref. Fukui, Sabae-shi, Kamikochi-cho, alt. 200 m, 25 October 2004, T. Sugawara 4102501 (C2-012766 HYO);
	Honshu, Pref. Gifu, Mugi-gun, Itadori-cho, along the Kaoredani valley (the upper stream of Itadori-gawa River), alt. ca. 500 m, 5 November 1994, Hiroshi Takahashi 15239 (C2-012776 HYO);
	Honshu, Pref. Fukui, Ono-gun, Izumi-mura, Tenguiwa, 35°55'00"N, 136°42'00"E, alt. 500 m, 22 October 1990, N. Kurosaki 17780 (C2-012772 HYO);
	Honshu, Pref. Fukui, Nanjo-gun, Kono-mura, Jindo, alt. 150–250 m, 20 September 1980, N. Kurosaki 11008 (C2-012758 HYO);
	Honshu, Pref. Gifu, Gujo-gun, Shirotori-cho, Itoshiro, along the Itoshiro-gawa River, alt. ca. 950 m, 30 September 2001, H. Takahashi 20839 (C2-012775 HYO);
	Honshu, Pref. Fukui, Ono-shi, On the border of Etsubi, Nukumi-toge pass, 24 September 1989, S. Iwatani 89092402 (C2-012771 HYO);
	Honshu, Pref. Fukui, Ono-shi, Houkyouji, 35°55' 00"N, 136° 28'00"E, alt. 450–600 m, 21 October 1990, N. Kurosaki 17721 (C2-012769 HYO);
	Honshu, Pref. Ishikawa, Mt. Haku-san, alt. 1500–2100 m, 28 August 1965, M. Hashimoto 4035 (C1-067764 HYO).
**S. glabrescens var. repens** * (Koidz.) S.Kurosaki	Honshu, Pref. Nara, Yoshino-gun, Totsukawa-mura, Mt. Tamaki-san, alt. 700–1000 m, 11 October 1975, N. Kurosaki 14015 (C1-012139 HYO);
	Honshu, Pref. Nara, Yoshino-gun, Totsukawa-mura, Mt. Tamaki-san, alt. 700–1000 m, 11 October 1975, N. Kurosaki 14045 (C1-012140 HYO);
	Honshu, Pref. Nara, Yoshino-gun, Totsukawa-mura, Mt. Tamaki-san, alt. 700–1000 m, 11 October 1975, N. Kurosaki 14045 (C1-012141 HYO);
	Honshu, Pref. Shiga, Koga-gun, Shigaraki-cho, Mt. Hando-san, alt. 500–600 m, 12 October 1975, N. Kurosaki 14048 (C1-012129 HYO);
	Honshu, Pref. Shiga, Koga-gun, Shigaraki-cho, Mt. Hando-san, alt. 500–600 m, 12 October 1975, N. Kurosaki 14047 (C1-012125 HYO);
	Honshu, Pref. Nara, Yoshino-gun, Totsukawa-mura, Mt. Tamaki-san, alt. 1000 m, 25 September 1994, A. Takahashi & T. Takahashi 2581 (C1-077729 HYO).
***S. nipponica*** Miq.	Honshu, Pref. Yamaguchi, Abu-gun, Ato-cho, Tokusa, 3 November 1968, Nagato Miake s. n. (C2-012360 HYO);
	Honshu, Pref. Fukui, Nyu-gun, Ota-cho, en route from Yotsusugi to Mt. Rokusho-san, alt. 300–650 m, 16 October 1975, N. Kurosaki 7015 (C2-012757 HYO);
	Honshu, Pref. Fukui, Nyu-gun, Mt. Ochi-san, alt. 200–600 m, 4 November 1971, N. Kurosaki 4646 (C2-012751 HYO);
***S. nipponica*** Miq.	Honshu, Pref. Hyogo, Shiso-gun, Haga-cho, Onzui, 35°14'00"N, 134°31'00"E, alt. 500–650 m, 17 October 1987, N. Kurosaki & Y. Takita 381 (C2-012448 HYO);
	Honshu, Pref. Hyogo, Shiso-gun, Chigusa-cho, Nabegatani, 23 September 1986, Tomiki Kobayashi 06575 (C2-012432 HYO).
***S. plebeia*** R. Br.	**Sect. *Notiosphace* Benth**.
	Honshu, Pref. Osaka, Takatsuki-shi, Udono, right side river bed of Yodo-gawa River, 34°51'30"N, 135°39'30"E, alt. 10 m, 6 June 1996, T. Umehara 7195 (C2-012814 HYO);
	Honshu, Pref. Osaka, Takatsuki-shi, Otsuka 2 chome, right side river bed of Yodo-gawa River, 34°49'00"N, 135°38'30"E, alt. 9 m, 25 June 1992, T. Umehara 4735 (C2-012815 HYO);
	Honshu, Pref. Okayama, Okayama-shi, Kawase, Asahikawa, 34°45'00"N, 133°55'00"E, alt. 50–100 m, 10 June 2001, T. Kobayashi 36018 (C2-012816 HYO);
	Kyushu (Ishigaki-jima Island), Pref. Okinawa, Ishigaki-shi, Hirakubo, Kuura, 24°33'00"N, 124°17'00"E, alt. 20–50 m, 11 May 2000, N. Fukuoka & N. Kurosaki 12103 (C2-012819 HYO);
	Kyushu (Ishigaki-jima Island), Pref. Okinawa, Ishigaki-shi, Yashima-cho, Southwest of Ishigaki Airport, 24°19'30"N, 124°10'00"E, alt. 4–5 m, 1 March 2002, Koji Yonekura 7706 (C2-012820 HYO).

## Additional information

### Artificial Intelligence (AI) use

The authors accept full responsibility for the content of the manuscript, including the disclosure of any use of AI.

AI tools were used in the preparation of this manuscript.

**Used for**: academic English editing.

### Author ORCIDs

A. Khan https://orcid.org/0000-0002-5337-7237

A. Takano https://orcid.org/0000-0002-8345-5080

### Data availability

All of the data that support the findings of this study are available in the main text.
